# A Controlled Simulation Study of Degeneracy-Aware Pose Fusion in Confined Metal Compartments

**DOI:** 10.3390/s26216873

**Published:** 2026-10-30

**Authors:** Jiexin Wang, Lei Li, Xiaoyu Jia, Qing Liu

**Affiliations:** School of Mechanical Engineering, Jiangsu University of Science and Technology, Zhenjiang 212100, China; 241210201322@stu.just.edu.cn (J.W.);

**Keywords:** pose fusion, degeneracy-aware estimation, controlled simulation, adaptive observation weighting, conditional registration, confined metal compartments

## Abstract

Geometric degeneracy and poor illumination make localization difficult in confined metal compartments. This study examines quality-dependent pose fusion in a four-dimensional position-and-yaw model using known-map LiDAR registration, synthetic visual poses, and noisy velocity and yaw-rate inputs. A geometric-visual quality score controls the combination of iterative closest point (ICP) and normal distributions transform (NDT) pose observations, while modality-specific covariance scales and innovation checks regulate state updates. Across 60 controlled Monte Carlo trials, the full configuration achieves a mean position error of 0.029 m, a between-trial standard deviation of 0.003 m, a mean position root-mean-square error of 0.034 m, and a mean peak error of 0.112 m. Frames in error runs above 0.15 m lasting at least five samples account for 0.00%. Representative processing time is 25.65 ms per frame, excluding synthetic generation. Paired ablations identify LiDAR innovation screening as the main measured accuracy contributor; independent mean-accuracy benefits from covariance and mixing adaptation are not established. Conditional NDT reduces registration work. Sustained common pose-observation corruption raises the mean error from 0.066 m at 6 s to 0.178 m at 24 s, and a fixed reduced configuration remains competitive. A separate field point-cloud study evaluates compact map representation. These findings characterize controlled pose-fusion behavior; sensor-level LiDAR-visual-inertial performance and real-compartment trajectory accuracy require independently referenced validation.

## 1. Introduction

Robots inspecting ship compartments and ballast tanks need reliable pose estimates. Large metal planes, repeated stiffeners, weak texture, poor lighting, and reflections can degrade light detection and ranging (LiDAR) registration and visual tracking [1,2,3,4]. This study examines how pose fusion responds when the quality of these observations changes.

Direct LiDAR-inertial odometry (LIO) reduces reliance on hand-crafted features and supports efficient state estimation [5,6]. LiDAR-visual-inertial (LVI) systems add visual constraints to geometric information [7,8,9,10,11,12]. R3LIVE, R3LIVE++, VILENS, and FAST-LIVO2 use different tightly coupled combinations of LiDAR, camera, and inertial measurements [8,9,10,11]. Their complementary use of sensing channels motivates the observation-quality problem examined here.

The available constraints change as the robot moves. Large planes and repeated structures can make registration ill-conditioned. Underexposure, directional lighting, saturation, and specular reflections can violate the assumptions of direct visual alignment [9,10,13,14,15]. Robust registration and radiometric modeling address different causes of degradation. The fusion policy must also decide which observations to use, how strongly to weight them, and when to reject a correction or attempt recovery.

Filter-based LVI is attractive for resource-constrained inspection because it does not repeatedly optimize a long state history [5,10]. An incorrectly weighted observation can still affect later estimates. We isolate this issue at the pose-observation level. The evaluated model does not perform raw inertial measurement unit (IMU) mechanization, image-based pose estimation, or registration against a map updated from estimated poses. It is not a complete LVI implementation.

This study makes two contributions within the controlled model. First, paired mechanism, stress, and transfer tests distinguish the effects of observation screening from covariance and mixing adaptation, including conditions in which added components provide little benefit. Second, a paired computational comparison evaluates conditional NDT scheduling with voxel-statistics reuse. Together, these experiments characterize the accuracy and computational trade-offs of quality-dependent pose fusion.

## 2. Related Work

### 2.1. Direct LiDAR and LiDAR-Inertial State Estimation

Direct LIO registers raw points or local statistical structures and combines the resulting constraints with inertial propagation. FAST-LIO2 supports high-rate estimation through direct raw-point registration and an efficient map structure, without a separate feature-extraction front end [5]. Long corridors, large planes, and repeated structures can still leave some motion directions poorly constrained [14,15]. Field analysis shows why degeneracy mitigation should preserve reliable directions while limiting unsupported updates along weak ones [16].

Degeneracy detection is closely related to uncertainty modeling. Pose covariance on the three-dimensional rigid-motion group SE(3) requires a consistent perturbation convention [17]. LOG-LIO2 propagates range, incidence-angle, and surface uncertainty into state estimation [18]. Our study examines a narrower issue: adapting observation weights during pose updates as observation quality changes.

### 2.2. Tightly Coupled LiDAR-Visual-Inertial Measurement

Tightly coupled systems combine sensor constraints in one estimator. LIO-SAM, LVI-SAM, and VILENS use graph-based formulations [7,11,19]. R3LIVE and FAST-LIVO2 use filter-based coupling and direct visual observations [8,10], and R3LIVE++ also models exposure and radiance [9]. These systems exploit complementary measurements, but adding a sensor does not ensure that it provides useful information in every scene.

In confined metal compartments, LiDAR and vision may degrade separately or together. Fixed modality weights can then give a poor observation too much influence. The method uses separate geometric and visual indicators and retains a separate covariance scale for each channel. The unified score coordinates the registration mixture; it does not replace the channel-specific checks.

### 2.3. Degeneracy Detection and Mitigation

Zhang et al. [20] separate weakly constrained directions from well-conditioned ones and restrict optimization updates in the weak directions. Their analysis motivates directional protection. Our reduced-order implementation attenuates selected update components.

Degeneracy-aware methods examine Hessian spectra, constrain weak directions, adapt registration objectives, or switch to an auxiliary modality [13,14,15,16,21]. TEASER++ provides robust rigid-transform initialization under substantial correspondence contamination [22] and represents a relevant alternative for recovery from severe registration failure. The present implementation instead uses a bounded position-and-yaw search over a local portion of the known simulation map. Its acceptance checks do not carry a certifiable-registration guarantee.

A binary switch may change state repeatedly when a diagnostic fluctuates near its threshold. Continuous weighting and hysteresis can reduce this behavior. Neither guarantees statistical consistency or restores missing constraints. Pose uncertainty representations [17] and the point-uncertainty model in LOG-LIO2 [18] address related modeling issues. Here, covariance inflation controls observation weights; its effects are assessed through repeated controlled trials.

### 2.4. Measurement Reliability and Illumination Robustness

Exposure changes, shadows, occlusion, and specular surfaces affect direct visual alignment. Existing systems use radiometric correction, exposure estimation, and robust residuals to address these effects [9,10]. In our experiments, synthetic images provide quality diagnostics, while synthetic poses provide visual filter observations. Appendix A describes a possible pixel-level extension that is not executed in this study.

Metrological guidance distinguishes a measurement result from its uncertainty [23,24]. A filter covariance used to weight updates is not automatically a traceable uncertainty budget. We use the synthetic reference to evaluate position error and the field-measured point cloud to examine map representation. Point-to-centroid distance measures the approximation introduced by voxelization, not localization error.

### 2.5. Research Gap and Scope

Shared known-map registration outputs and synthetic visual poses allow fusion variants to receive controlled inputs. This design separates observation-weighting effects from image tracking and map feedback. It supports a mechanism comparison, not a benchmark of complete LVI systems.

## 3. Method

### 3.1. System Overview

#### 3.1.1. Measurement Scenario and Problem Definition

The target setting is a confined metal compartment with bulkheads, stiffeners, longitudinal passages, and pipework. Its geometry constrains some motion directions more strongly than others. Limited lighting and specular surfaces can also reduce visual quality. The implemented state estimates three-dimensional position and yaw; these are the variables evaluated in the experiments.

The model combines noisy velocity and yaw-rate inputs, scans registered against a known map, and synthetic visual poses. Section 3.1.2 defines the state and inputs before the update pipeline is introduced.

Section 3.1, Section 3.2, Section 3.3 and Section 3.4 describe the implemented algorithm. The pixel-level design in Appendix A is not implemented.

#### 3.1.2. State Variables and Sensor Inputs

Equation (1) defines the estimated state at sample k. The first three entries are Cartesian position in the fixed simulation frame, and the fourth is yaw about its vertical axis, in radians. Position changes in three dimensions, but roll and pitch are not estimated. A sample interval of 0.08 s is used. The common initial pose is supplied explicitly; subsequent reference poses are available only to the simulator and evaluator.

(1) $$x_{k}=[x_{k},y_{k},z_{k},\psi_{k}{]}^{⊤}$$

Velocity and inertial biases are not estimated. All variants start from the same supplied pose. Later reference poses are used only to generate data and evaluate results, never to correct the estimate, gate an observation, or guide recovery. Section 4.10.1 discusses extension to full motion and inertial bias estimation.

The reference trajectory is generated by sampling a prescribed three-dimensional waypoint polyline, adding bounded sinusoidal position perturbations, and keeping the path inside the compartment. Yaw follows the horizontal path direction, is smoothed, and includes a prescribed scan oscillation. Body-frame reference velocity is obtained from successive positions using the previous reference yaw; reference yaw rate is the wrapped yaw difference divided by the sample interval. The simulator then adds independent zero-mean Gaussian input noise with standard deviations of 0.025 m/s in each velocity component and 0.015 rad/s in yaw rate. This controlled input model does not include estimated accelerometer or gyroscope biases.

LiDAR observations come from iterative closest point (ICP) and normal distributions transform (NDT) registration of ray-cast scans against the fixed known map. Scan generation includes 0.010 m range noise and 0.03-degree angular noise, with scene-dependent dropout and reflective outliers specified in the configuration. The registration observations are computed before filter replay and then shared across compared fusion variants. Their errors arise from that front end; the synthetic LiDAR pose generator is not substituted for failed registrations in this protocol.

The visual generator adds a score-dependent bias, component-wise Gaussian noise, and a compound-segment outlier to the reference pose, wrapping yaw afterward. For the raw proxy, the standard-deviation vector is (0.060 m, 0.060 m, 0.045 m, 1.8 degrees) multiplied by one plus 4.3 times the visual score. For the high-dynamic-range (HDR)-equivalent proxy it is (0.052 m, 0.052 m, 0.040 m, 1.5 degrees) multiplied by one plus 1.45 times that score. The raw bias is (−0.10 m, 0.08 m, −0.020 m, −1.4 degrees) multiplied by the squared visual score; the HDR-equivalent bias is 0.35 times this vector. Angles are converted to radians internally.

In a compound segment, an outlier is drawn with probability 0.07. Its components are independent uniform values in symmetric intervals with half-widths (0.35 m, 0.25 m, 0.09 m, 4 degrees); the raw and HDR-equivalent proxies receive 0.6 and 0.25 times the same draw, respectively. Their Gaussian draws are separate. These assumed error distributions encode a visual-quality hypothesis; they do not demonstrate that a radiometric optimizer achieves the stipulated improvement. The reference is confined to measurement generation and evaluation, and is not available to the filter correction, gating, or recovery logic.

Propagation rotates the measured body-frame velocity using the previous estimated yaw. Multiplying by the sample interval gives the displacement added to the previous position. Yaw is advanced using the measured yaw rate and wrapped to the principal interval. The transition Jacobian is the four-dimensional identity, with a position-versus-yaw column given by the yaw derivative of the rotated displacement. The previous covariance is propagated through this Jacobian, then the process covariance is added. The functions motionModel3D and run3DFusionFilter implement these steps; they do not perform raw inertial mechanization.

The initial working covariance is diagonal with squared entries based on standard deviations (0.08 m, 0.08 m, 0.06 m, 3 degrees). The per-step base process covariance similarly uses (0.020 m, 0.020 m, 0.015 m, 0.8 degrees), and is multiplied by the configured causal process scale when adaptation is enabled. It is an estimator design covariance, not a covariance derived solely from the input-noise values above. Pose-observation innovations subtract the predicted position and wrap the yaw difference; their observation Jacobian is the identity. These definitions complete the implemented motion and observation interface before Algorithm 1.

The implemented outputs are:

- The position-and-yaw estimate and its working filter covariance;
- Framewise registration, gating, overlap, and recovery diagnostics;
- Quality-qualified keyframe-candidate records used by the consistency safeguard.

Candidate records contain quality and novelty information but do not alter the registration map. This fixed map experiment cannot test whether the admission policy prevents map contamination.

#### 3.1.3. Measurement Pipeline

**Figure 1:**
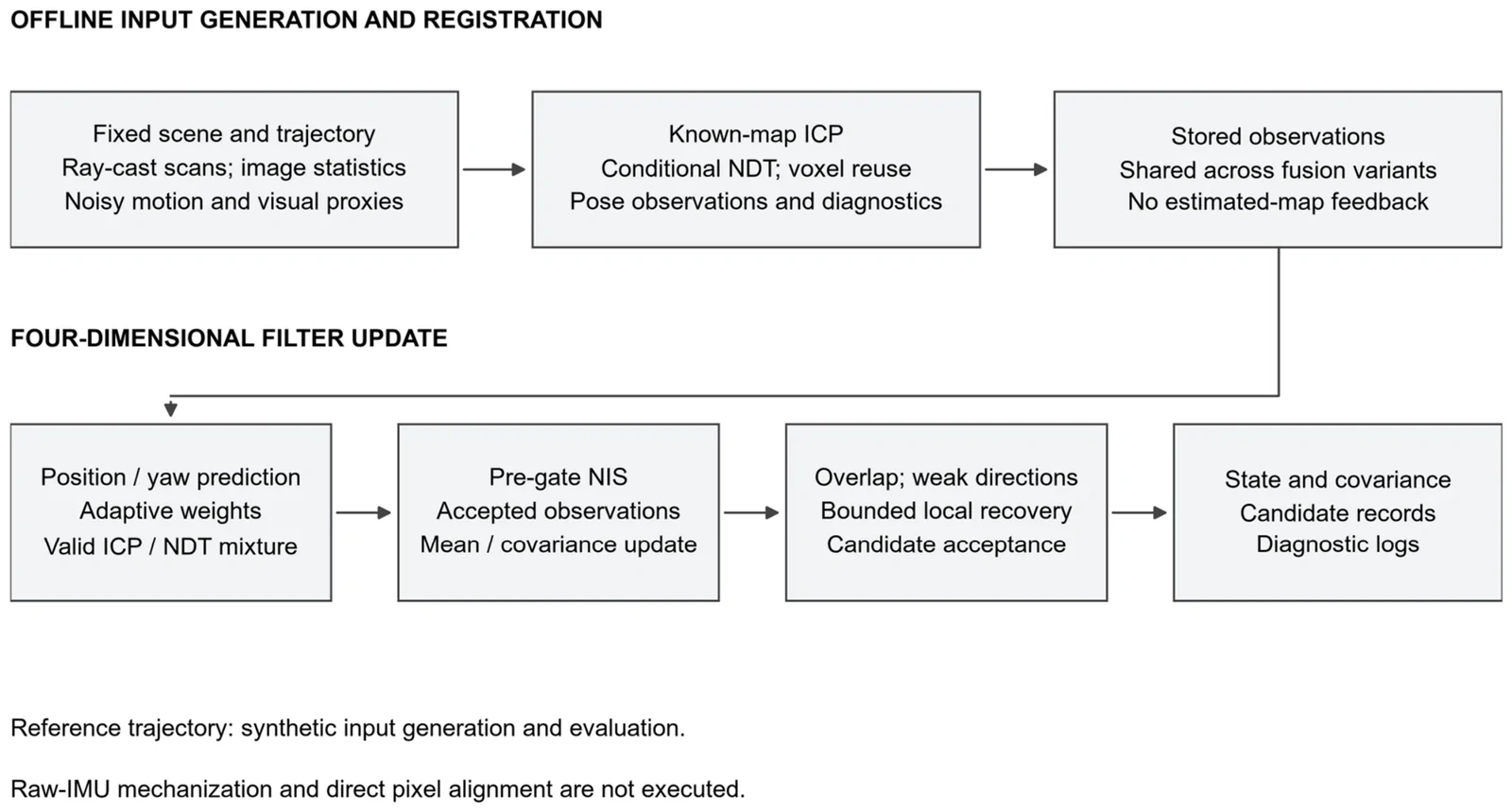
The data flow of the controlled degeneracy-aware pose-fusion framework, including precomputed LiDAR registration, visual quality evaluation, adaptive observation weighting, gated state correction, and local recovery.

Figure 1 shows the executed data flow. Synthetic scans and images are generated offline. ICP runs first. Its residual, conditioning, correspondence count, and overlap, together with a scan-geometric indicator, determine whether NDT is needed. The final geometric score uses the valid registration Hessians available after this decision. NDT activation does not depend on a Hessian that has yet to be computed.

The filter predicts the four-dimensional state and combines valid ICP/NDT pose observations in a shared tangent coordinate system. Covariance scaling and innovation checks determine whether and how strongly each observation corrects the state. Vision supplies a synthetic raw or high-dynamic-range (HDR)-equivalent pose. Image statistics determine its confidence; they do not solve for the visual pose.

In the full configuration, overlap and weak-direction diagnostics also regulate updates. Observable anomalies can trigger a bounded local recovery search. A candidate is accepted only if it improves geometric cost and visual compatibility and passes the overlap check. The accepted correction is limited in size. Candidate keyframe records are stored after this decision. The known registration map remains fixed, so corrected poses do not feed back into an evolving point map.

Algorithm 1 gives the executed update order.
**Algorithm 1.** Degeneracy-aware low-compute pose update in the controlled LVI mechanism modelInput: stored LiDAR registration observations and local scan, synthetic image-quality diagnostics and visual pose observation, noisy velocity/yaw-rate prediction, known scene map. Output: position-and-yaw state, working covariance, candidate-keyframe record, and diagnostic flags. 1. Propagate position, yaw, and covariance using the noisy velocity and yaw-rate inputs. 2. Read valid ICP/NDT observations from the precomputed front-end pass, which uses hysteretic NDT activation and voxel-statistics reuse. 3. Compute geometric information shape and image-quality scores; update the unified score and nominal sigmoid mixing weight causally. 4. Form a common four-dimensional observation from valid ICP/NDT estimates. Skip the LiDAR update if both are invalid. 5. Apply modality-specific covariance scaling, then compute pre-gate normalized innovation squared (NIS) using predicted covariance. 6. Accept, soften, or reject the LiDAR update. Apply the same damped effective gain to the mean and Joseph covariance update. 7. Compute visual confidence and pre-gate NIS. Reject low-confidence or hard-gated visual updates; log enabled, accepted, and rejected separately. 8. Check local overlap, structure diversity, motion novelty, and innovation. Treat candidate admission as a quality record, not an updated point map. 9. Update the normalized recovery score and consecutive count. If threshold, cooldown, and budget permit, evaluate a bounded local-search candidate. 10. Accept a small recovery step only if the cost and visual-compatibility ratios pass their fixed thresholds and overlap satisfies the nondegradation rule; otherwise retain the pose and inflate working covariance. 11. Commit an admissible candidate record, save actual covariances and diagnostics, and continue. The known registration map remains fixed.

Table 1 lists key defaults, adaptive ranges, and evaluation-only thresholds.

Table 1 reports shared engineering defaults and bounded adaptation ranges. The geometric weights 0.70/0.30 give more emphasis to conditioning than to the minimum-eigenvalue indicator. The visual weights 0.45/0.20/0.25/0.10 emphasize gradient deficiency and saturation, with smaller contributions from darkness and contrast. These weights were not selected using an independent calibration dataset.

Normalized innovation squared (NIS) thresholds are based on four-dimensional Gaussian reference quantiles. Adaptive working covariances mean that nominal rejection probabilities cannot be assumed. Clipping, smoothing, and recovery budgets limit parameter changes and correction cost. Section 4.8 reports the available single-sequence sensitivity analysis.

### 3.2. Degeneracy-Aware Observation Adaptation

The policy uses observation quality in two ways. The mixing weight controls the relative contribution of available registration estimates. The covariance scale controls how strongly each observation channel changes the state. The scores guide weighting and registration decisions; they are not calibrated error probabilities.

The module addresses two questions:

- How can geometric and photometric degradation be summarized together?
- How should that summary guide registration and filter updates when some constraints become weak?

Geometric and image-quality indicators are converted to bounded scores. The unified score controls the nominal registration mixture. Separate modality scores control covariance inflation, so the same unified score is not applied as an identical noise multiplier to both channels.

#### 3.2.1. Unified Degeneracy Score

The geometric indicator describes which directions local registration constrains. The visual indicator summarizes gradient, illumination, saturation, and contrast statistics.

Equation (2) defines the raw Gauss–Newton information approximation for each registration objective. The index i identifies a correspondence, $J_{i}$ is its residual Jacobian with respect to the four pose coordinates, $W_{i}$ is its weighting matrix, and the superscript ⊤ denotes transpose. This approximation describes local registration information, not global pose observability.

(2) $$H_{L}=\sum_{i}J_{i}^{⊤}W_{i}J_{i}$$

The ICP and NDT Hessians are processed separately. For either registration method j, define $A_{j}{=\;T}^{⊤}{(H}_{j}{+H}_{j}^{⊤})T/2$, with T= diag(1,1,1,1/l) and characteristic length l = 1 m. Each valid matrix is trace-normalized as ${\overset{-}{H}}_{j}{=\;A}_{j}{/\mathrm{tr}(A}_{j})$. The diagnostic matrix G is the sum of the valid ICP information shape and 0.35 times the valid NDT information shape; unavailable terms are omitted. No second trace normalization is applied. Equation (3) uses the spectrum of G to obtain the raw geometric score.

(3) $${S_{L}}^{\mathrm{raw}}=0.70{\mathrm{norm}}_{c}(\mathrm{cond}(G))+0.30{\mathrm{norm}}_{l}(-\log_{10}\lambda_{\min}\left(G)\right)$$

The bounded normalizations use the 20th and 95th percentiles of available past and current diagnostics, with fixed fallback limits before sufficient history exists. This cumulative normalization differs from the 40-frame parameter-controller windows. Conditioning is capped at $10^{12}$, and eigenvalues are floored at $10^{-12}$ only for logarithms. If both registration methods fail, the measurement-generation path instead assigns $S_{L}^{\mathrm{raw}}\;=\;\max(S_{L,\mathrm{scan}},0.85)$, where $S_{L,\mathrm{scan}}$ is the scan-geometric indicator. The information helper returns 1 when it has no valid information matrix; this is a separate case, not the rule for all double-registration failures.

In Equation (3), cond is the spectral condition number, $\lambda_{\min}$ is the minimum eigenvalue of G, and ${\mathrm{norm}}_{c}$ and ${\mathrm{norm}}_{l}$ are the respective bounded causal normalizations. The coordinate scaling sets the translation-versus-yaw convention. It does not make the score invariant to all sensor models, scene scales, or correspondence weights.

For a normalized image, mean gradient magnitude is defined in Equation (4).

(4) $$E_{g}=\frac{1}{\left|\Omega \right|}\sum_{u\in \Omega }\|\nabla \;I(u){\|}_{2}$$

In Equation (4), I is an intensity-normalized image, u is a pixel, Omega is the image region used for quality evaluation, and its cardinality is the pixel count. The gradient operator takes image-plane intensity derivatives and the norm is Euclidean. Equation (5) combines four bounded deficiencies: *d_g_* for gradient, *d_dark_* for dark pixels, *d_sat_* for saturated pixels, and *d_c_* for contrast. Larger values indicate poorer image quality, not a directly measured pose-error variance.

(5) $${S_{V}}^{\mathrm{raw}}=0.45d_{g}+0.20d_{\mathrm{dark}}+0.25d_{\mathrm{sat}}+0.10d_{c}$$

Dark and saturated pixels use intensity cutoffs of 0.08 and 0.95. Gradient and contrast references use causal 95th-percentile histories after ten samples, with initial references 0.075 and 0.18. Equation (5) gives the raw visual score. Before Equation (6), each raw geometric and visual score is averaged over the current frame and at most four preceding frames; at sequence start, only available frames are used. Thus $S_{L}$ and $S_{V}$ in the fusion and covariance controllers are five-frame causal averages, not the unsmoothed scores. Innovation screening remains a separate filter check.

Equation (6) combines the geometric and visual scores using a bounded, causally adapted weight.

(6) $$S\;=\alpha \;S_{L}+(1\;-\;\alpha )S_{V}$$

The weight alpha starts at 0.55. Its instantaneous target is the geometric reliability divided by the sum of geometric and visual reliability, where each reliability is one minus its bounded score. If both reliabilities vanish, the default is retained as the target. The controller combines 85% of the previous weight with 15% of this target and clips the result to [0.25, 0.75]. This is the rule implemented in computeAdaptiveAlpha. Because a severely degraded channel can receive a smaller weight in this average, the fused score need not represent the worst channel. Separate modality scores and validity checks remain necessary; the fused score is not a failure probability.

#### 3.2.2. Degeneracy-Aware Registration Observation Adaptation

Point-to-plane ICP and NDT solve separate registration problems and return poses in the same position-and-yaw space. The point residuals below explain the geometry behind each method. The filter combines their pose observations, not their point residuals element by element.

For a transformed source point and its associated local plane, Equation (7) defines the scalar point-to-plane residual.

(7) $$r_{p,i}=n_{i}^{⊤}(Rp_{i}+t-q_{i})$$

In Equation (7), *p_i_* is a source scan point, *q_i_* is its associated map-plane point, and *n_i_* is the unit plane normal. R and t rotate and translate the source into the fixed map frame; R is a yaw rotation in the evaluated model. The scalar residual measures displacement along the normal in meters. Repeated observations of a single plane do not resolve translation along that plane.

For NDT, Equation (8) defines a whitened displacement to a local Gaussian cell.

(8) $$r_{n,i}=\Sigma_{v(i)}^{-1/2}(Rp_{i}+t-\mu_{v(i)})$$

In Equation (8), v(i) identifies the destination voxel, mu is its point mean, and Sigma is its regularized three-dimensional covariance. The inverse square root whitens the displacement. This residual is a three-component dimensionless vector, whereas Equation (7) is scalar and measured in meters. They are not added directly. NDT can itself fail under low occupancy, poor overlap, or insufficient structural diversity.

Both valid pose innovations are measured relative to the same predicted state, with the same yaw-wrapping convention. Equations (9) and (10) use a covariance-intersection rule [25] to combine these four-dimensional innovations and their working covariances. They are different quantities from the point residuals in Equations (7) and (8).

(9) $$R_{c}=[(1\;-\;\beta )R_{p}^{-1}+\beta R_{n}^{-1}{]}^{-1}$$

(10) $$r_{c}=R_{c}[(1\;-\;\beta )R_{p}^{-1}r_{p}+\beta R_{n}^{-1}r_{n}]$$

In Equations (9) and (10), *R_p_* and *R_n_* are four-dimensional working pose covariances from ICP and NDT; *r_p_* and *r_n_* are innovations relative to the same predicted pose, with yaw differences wrapped consistently. *R_c_* and *r_c_* denote the combined covariance and innovation. They are not the scalar or three-component point residuals above. Equation (11) uses *k_β_* as the sigmoid slope and *τ_β_* as its midpoint, so increasing the unified score increases the nominal NDT contribution.

(11) $$\beta (S)=\frac{1}{1+\exp[-k_{\beta }(S\;-\tau_{\beta })]}$$

The effective mixing weight is zero if only ICP is valid and one if only NDT is valid. If both fail, the LiDAR update is skipped. No reference pose is substituted for failed registration. Conditional NDT activation and its hysteresis are separate decisions from the nominal smooth mixing weight.

ICP and NDT observations can be correlated because they use related data. Their cross-correlation is not explicitly modeled, which motivates covariance intersection [25]. The combined covariance is used for recursive weighting, not reported as calibrated posterior uncertainty. Combining identical inputs leaves their covariance unchanged rather than halving it.

Covariance intersection does not make arbitrary input covariances conservative [25]. Its consistency interpretation requires valid input error bounds. The registration covariances here have not been calibrated to provide those bounds, so the combined matrix remains a working weight rather than a guaranteed error bound.

#### 3.2.3. Observation-Quality-Driven Covariance Scaling

The observation covariances determine the relative strength of LiDAR and visual corrections during recursive fusion. The modality-specific scaling law is given in Equation (12).

The registration observations are combined first. Their LiDAR covariance is then scaled once by the geometric score. Vision uses its own image-quality score and confidence. Base scales change slowly using past innovation statistics.

(12) $$R_{L}=R_{c}(1+k_{L}S_{L}^{\gamma_{L}}),\;R_{V}=R_{V}^{0}s_{V}(1+k_{V}S_{V}^{\gamma_{V}})$$

In Equation (12), kL and kV control the amplitude of score-based inflation and γL and γV control its curvature. RV0 is the selected raw or HDR-equivalent base visual covariance; sV collects the separate visual scale factors. Table 1 gives defaults and bounds, and Supplementary Table S2 gives base covariances. Inflation reduces observation gain but does not calibrate an inaccurate noise model. Section 4.8.1 evaluates the covariance adaptations as a group.

In Equation (13), P-minus is the predicted state covariance, H is the observation Jacobian, and *R_used_* is the covariance actually used after accepted inflation. For the executed pose observations, H is the four-dimensional identity. The Kalman gain K maps an innovation into a state correction.

(13) $$K\;=P^{-}H^{⊤}{\left(HP^{-}H^{⊤}+R_{\mathrm{used}}\right)}^{-1}$$

When weak-direction damping is enabled, the effective gain is DK. The same effective gain is used for both the state correction and the Joseph covariance update in Equations (14) and (15). This prevents applying a damped mean correction with an undamped covariance reduction. The matrix used in the Joseph term includes any gate and Huber inflation actually applied to the observation.

In Equations (14) and (15), D is the direction-dependent damping operator, *K_e_* is the effective gain, delta x is the state correction, I is the identity, and P-plus is the updated working covariance. Without damping D is the identity. Matching the mean and covariance algebra does not by itself establish unbiased estimation or statistical consistency.

(14) $$K_{e}=\;DK,\;\delta x=K_{e}r$$

(15) $$P^{+}=(I-K_{e}H)P^{-}{\left(I-K_{e}H\right)}^{⊤}+K_{e}R_{\mathrm{used}}K_{e}^{⊤}$$

For an innovation r, the pre-gate innovation covariance is $V\;=\;HP^{-}H^{⊤}+R_{\mathrm{pre}}$. The normalized innovation squared (NIS) is $r^{⊤}V^{-1}r$; the displayed innovation norm is $\sqrt{\mathrm{NIS}/4}$. Fixed four-dimensional chi-square reference cutoffs are 9.4877 and 13.2767. An observation above the upper cutoff is rejected; the intermediate region receives covariance inflation. No second test is used to accept a measurement solely because that same rejected innovation inflated its covariance. Because the covariance is adaptive and data-dependent, these cutoffs are engineering reference levels, not validated nominal false-alarm probabilities.

This is a four-dimensional extended Kalman filter (EKF)-style pose update, not a joint inertial error-state estimator on SE(3).

A rejected observation makes no direct correction to the mean state. If one channel is accepted, it corrects the prediction. If both are rejected, the filter continues prediction and checks the separate recovery conditions. Recovery is also rejected when no valid visual compatibility check is available, as specified in Section 3.4.2. Continuing prediction keeps the algorithm running, but error can grow when informative observations remain unavailable. The checks do not diagnose hardware fault types or faults in the propagation input.

The tested mechanism adapts pose observations using quality indicators, without reference-error feedback. Its evidence is limited to the known-map and synthetic visual model in Section 4.1.

### 3.3. Executed Visual Proxy and Innovation Screening

The visual channel supplies a synthetic four-dimensional pose, not a pose estimated from pixel residuals. Synthetic image statistics determine quality and confidence. The generator in Section 3.1.2 supplies the raw or high-dynamic-range (HDR)-equivalent pose. The HDR-equivalent label means that the generator assumes a lower-error distribution; it does not show that radiometric compensation achieves this reduction. Appendix A contains the proposed image-level equations, which are not used to explain the measured gains.

R3LIVE++ [9] combines photometric calibration with online exposure estimation. It motivates the prospective radiometric design in Appendix A, whose evaluation requires a calibrated pixel-level implementation and image-based experiments.

Executed visual confidence combines gradient confidence, exposure confidence, a contrast-derived patch-quality proxy, and a score-derived photometric-quality proxy, with weights 0.35, 0.25, 0.20, and 0.20. The patch proxy is not a measured patch count, and the photometric proxy is not a reprojection residual. Confidence below 0.18 rejects the visual observation when screening is enabled; intermediate confidence inflates its covariance. The base visual scale grows smoothly and recovers more slowly using coefficients 0.88 and 0.97, respectively. Enabled, accepted, and rejected states are logged separately, along with pre-gate and actually used covariance matrices.

The visual innovation uses the same position-and-yaw coordinates as the state. The gate asks whether an observation agrees with the prediction; it does not check image correspondences directly. It may reject a harmful correction, but an inaccurate prediction can also cause it to reject useful information. The original ablation shows no meaningful average-error benefit from the visual gate. Section 4.8.5 tests it under visual-only pose corruption, with confidence and validity checks retained. That test does not evaluate pixel-level alignment.

### 3.4. Efficient Implementation and Conditional Recovery

A compact state, conditional NDT, and a limited number of recovery attempts keep the evaluated recursion small. The measured MATLAB profile is reported in Section 4.7. It does not include the proposed pixel-level extension.

#### 3.4.1. Filter-Based Implementation

The estimator updates position and yaw sequentially from pose observations. It has no iterated error-state loop or inertial bias states. Covariance matrices are symmetrized after updates. Invalid matrices and registration outputs are rejected explicitly.

The tested recursion combines noisy velocity and yaw-rate propagation, stored known-map registration results, and gated visual pose proxies. Its low cost partly comes from this reduced representation and cannot be assumed for a full raw-sensor LVI system. Table S5 specifies the scan-score trigger, hold counter, input budgets, and voxel-cache rules. Conditional NDT and cache reuse operate within one pipeline. Section 4.10.1 and Section 4.10.3 discuss those extensions.

The filter avoids maintaining a long optimization window. Actual latency still depends on the software and hardware. The timings in Section 4.7 apply only to the reported MATLAB profiling conditions.

The local-consistency check measures overlap, directional diversity, planarity, and candidate novelty. Low overlap, large residuals, or inconsistent innovations can block admission to candidate history. Displacement, yaw change, view change, or structural gain can make a candidate sufficiently new. These records do not change the registration map. Testing map-contamination prevention would require a map built from estimated poses, compared with and without this admission policy.

#### 3.4.2. Local Fallback Under Extreme Degeneracy

The optional recovery routine attempts a bounded correction when poor observations persist. The following trigger and acceptance rules define its operation in the full configuration.

The recovery score combines geometric/visual concern, normalized residuals and innovations, ICP quality, available NDT quality, overlap loss, an abnormal-frame count, update magnitude, and a working-covariance trace term. Table S4 gives all 12 components, scales, weights, and unavailable-data rules. The covariance trace mixes position-and-yaw coordinates and is an engineering trigger, not a dimensionless uncertainty measure. Equation (16) defines recovery eligibility when the module is enabled:

(16) $$\begin{array}{c} q_{k}=\frac{\sum_{j\in \;A_{k}}w_{j}\min(c_{j,k},1)}{\sum_{j\in \;A_{k}}w_{j}}\\ \;T_{k}=\;(n_{k}\;\geq \;2)\wedge (d_{k}\;=\;0)\wedge (b_{k}>0) \end{array}$$

The score uses weights normalized over the available components. Here, $n_{k}$ counts consecutive frames with $q_{k}\;\geq \;0.60$, $d_{k}$ is the remaining cooldown, and $b_{k}$ is the remaining attempt budget. A below-threshold frame resets the count to zero. Cooldown is eight frames after acceptance and four after rejection; the sequence-level budget is four attempts. The score condition and an actual recovery attempt are logged separately. These values are fixed before evaluation and never depend on reference position error.

Recovery searches a bounded translation-and-yaw neighborhood against at most 450 local-map points and 120 scan points. The proposed correction is shortened to 45% and limited to 0.18 m and 2.5 degrees per accepted step. Acceptance requires a geometric cost ratio below 0.99, more than 40 correspondences, and visual compatibility no worse than 0.995 times its pre-recovery value. The overlap check permits either a gain of at least 0.02 or nondecreasing overlap with sufficient correspondences. Both poses use the same distance-based geometric cost and the same accepted visual observation; the latter is an internal consistency check, not independent validation. Recovery is rejected when no valid visual check exists.

A rejected candidate leaves the state unchanged and inflates the working covariance to lower trust. An accepted correction adds its outer product to the covariance; acceptance does not automatically reduce uncertainty. The trigger, attempt, decision, rejection reason, cooldown, cost, overlap, and point counts are logged for later analysis.

Section 4.8.7 evaluates recovery with paired enabled/disabled runs under pose-observation corruption. Candidate admission is recorded separately because the registration map remains fixed.

#### 3.4.3. Update Bounds and Computational Accounting

The update bounds describe what the code does, not whether the estimator converges. Damping uses the same scaled position-and-yaw coordinates as the information matrix. In its orthonormal eigenvector basis, weak-direction components are multiplied by 0.15 and the others by one. An orthogonal transformation preserves Euclidean norm, and factors in [0, 1] cannot increase that norm. The damped increment is therefore no larger than the original increment in these scaled coordinates. This statement does not apply to an unscaled mixture of meters and radians. It also does not imply lower position error: damping a useful correction can leave prediction bias in the estimate.

Covariance inflation also has a limited interpretation. For a fixed prediction covariance and linear observation model, multiplying a positive-definite observation covariance by a scalar of at least one reduces its inverse in the positive-semidefinite order. The observation then contributes no more information than before. This does not mean that every Kalman-gain entry decreases or that estimation error improves. Residual-dependent adaptation and heuristic damping need separate consistency tests. The working covariance is not a traceable uncertainty budget.

Recovery scales a candidate increment by 0.45 and limits each accepted step to 0.18 m in translation and 2.5 degrees in yaw. Four allowed attempts permit at most four accepted corrections. Their direct translation lengths sum to at most 0.72 m, and their absolute yaw increments to at most 10 degrees. These bounds apply only to the injected corrections. Later prediction and updates can amplify or offset their effects, so the bounds do not limit final error or the difference from a no-recovery trajectory. A finite budget does not guarantee successful recovery or establish worst-case execution time.

For mutually exclusive per-frame timers, average processing cost is the sum of the average base cost, NDT active fraction times mean matching cost conditional on activation, and voxel-build fraction times mean incremental build cost conditional on rebuilding. Base cost must include trigger checks, cache access, ICP, and filtering, including recovery, so no component is counted twice. This is an accounting identity, not a timing prediction. Activation can select harder frames, and rebuilding can coincide with activation; an independence assumption is unnecessary if conditional averages are measured. Comparing conditional and always-on policies therefore requires their own timer records. Section 4.7 and Section 4.8 report the measured timing comparisons.

## 4. Experiments and Discussion

### 4.1. Experimental Protocol and Evidence Levels

The MATLAB benchmark contains bulkheads, stiffeners, pipes, reflective regions, and geometric, photometric, and compound-degeneracy segments. The trajectory includes reversals, diagonal traverses, height changes, and yaw scans. Sixty trials use seeds 1001–1060, 760 frames per sequence, and a sampling interval of 0.08 s. The scene, trajectory difficulty, noise magnitudes, and error thresholds are unchanged in the corrected experiment.

The four-dimensional state is the same as Equation (1); three-dimensional motion does not imply six-degree-of-freedom estimation.

LiDAR observations are precomputed by registering scans against the known synthetic map, then replayed across fusion variants. A perturbation model generates visual poses. Noisy velocity and yaw rate replace raw-IMU mechanization. Sharing these inputs isolates the fusion mechanism from front-end and map-feedback effects.

Within each seed, all variants share the synthetic trajectory, raw scans, synthetic images, and a pre-generated pool of raw and HDR-equivalent visual pose proxies. Each configuration selects its prescribed visual stream. The two streams have different Gaussian draws and noise amplitudes, so this comparison includes an observation-model difference; it does not give every variant identical visual observations. Reference pose is used for measurement generation and evaluation, not filter correction or recovery. NDT input sampling uses geometry-only grids, not simulator material labels. However, the initial scan-geometric trigger uses simulator surface normals, and registration uses the known scene map. Table 2 records this controlled protocol. The site-acquired point clouds in Section 4.9 are a separate representation study, excluded from localization-accuracy and estimator-runtime comparisons.

### 4.2. Simulation Environment and Local Observations

**Figure 2:**
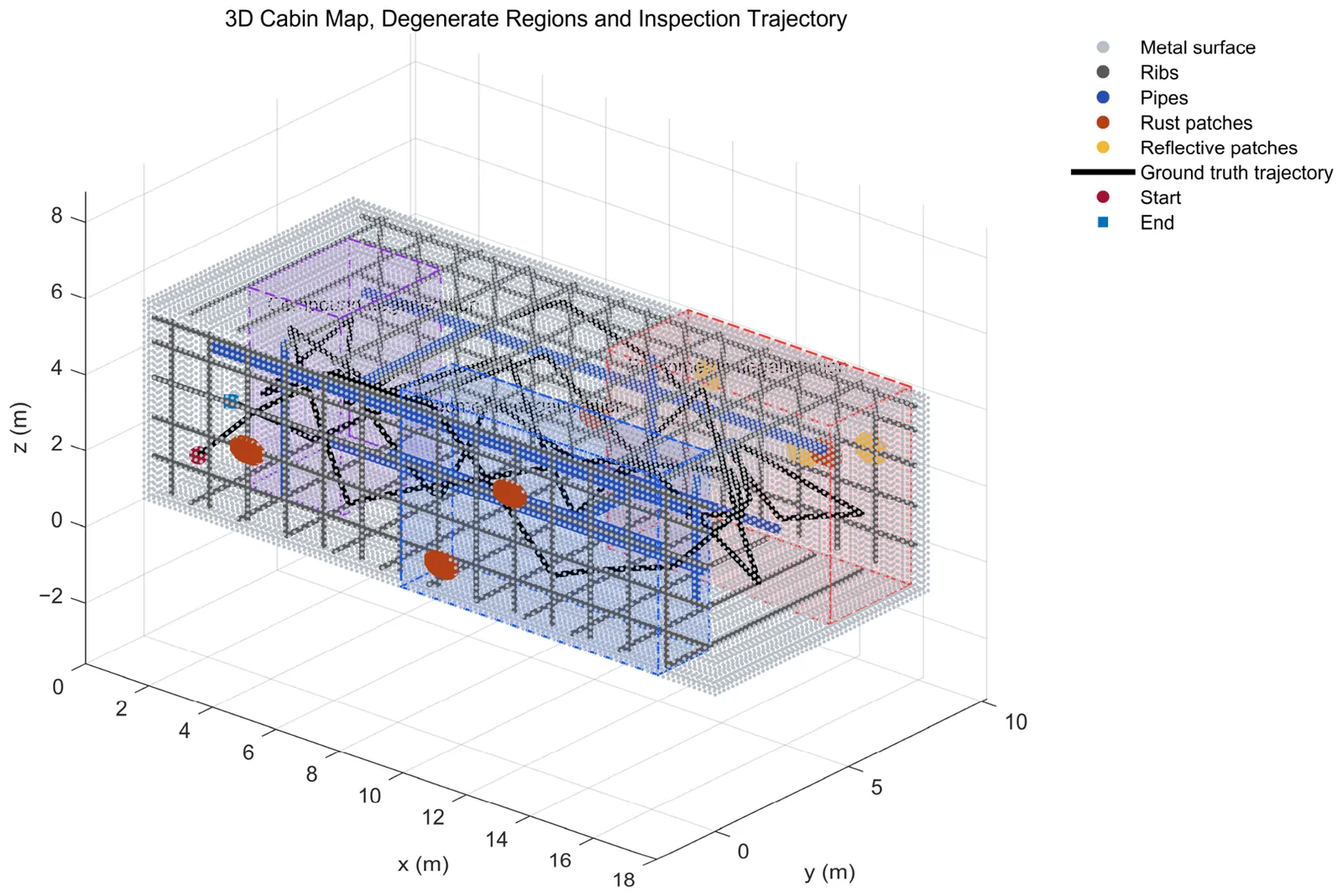
Simulated compartment, degenerate regions, and inspection trajectory. Blue, red, and purple wireframe boxes mark prescribed geometric, photometric, and compound degeneracy regions, respectively.

Figure 2 shows the 3-D compartment and inspection route. Stiffeners, pipes, and reflective regions provide different local structures and degradation conditions. The three-dimensional route changes LiDAR coverage and viewing direction as well as position, allowing these effects to be examined together.

**Figure 3:**
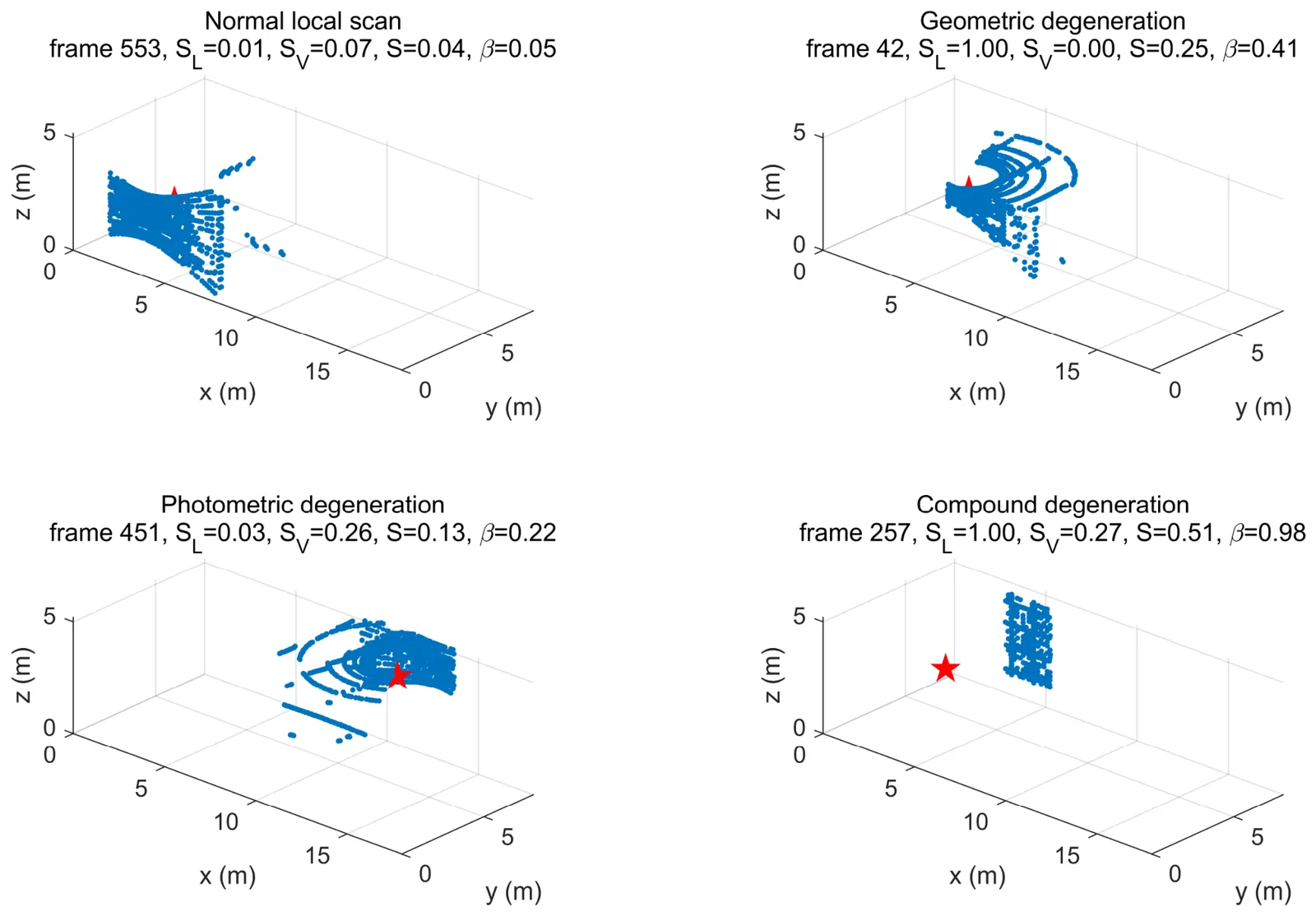
Representative local LiDAR scans under different degeneracy conditions. Red five-pointed stars mark the synthetic sensor reference position. Partial occlusion by blue scan points can make a star appear triangular; no separate triangle marker is used.

Figure 3 shows representative ray-cast local LiDAR scans under nominal, geometric-degeneracy, photometric-degradation, and compound-degeneracy conditions. The nominal scan covers structures in multiple directions and has a low geometric degeneracy score. Under geometric degeneracy, the scan is dominated by a single plane or repetitive structures and $S_{L}$ increases. During photometric degradation, visual quality decreases while geometry remains partially informative. The compound segment combines low overlap, weak geometry, and low visual confidence and is therefore the combined perturbation case within this controlled protocol.

### 4.3. Compared Variants and Measurement Metrics

FAST-LIO2-like and FAST-LIVO2-like are labels for controlled LiDAR-only and LiDAR-visual mechanism baselines, respectively. They are not executions, reproductions, or performance estimates of the official FAST-LIO2 [5] and FAST-LIVO2 [10] systems. The former uses LiDAR registration with the same reduced-order propagation inputs; the latter adds a synthetic visual pose proxy. +Mix+Cov enables pose-observation mixing and adaptive covariances, and +HDR selects an HDR-equivalent proxy rather than running image-based radiometric optimization. Ours-Full adds Huber weighting, LiDAR innovation screening, map-quality protection, weak-direction damping, and active recovery. Visual confidence weighting and the visual innovation gate are shared by visual variants unless explicitly disabled in internal ablation. Conditional NDT is a shared precomputed front-end policy. Thus, Table 3 compares configurations within one controlled model and cannot establish superiority over public-code LIO or LVI systems.

A sensor-level comparison should run the official FAST-LIO2 and a compatible visual-inertial system such as FAST-LIVO2 on the same calibrated recordings, with their sensor requirements satisfied. Versions, configurations, initialization, hardware, trajectory alignment, and incomplete runs should be reported. An enhanced version of the same public-code host would then isolate the added adaptation policy from differences in front-end design. Such executions are not included in the present results; Section 4.10.4 sets out the required validation pathway.

Position error is the Euclidean distance between the estimate and reference in the shared synthetic frame. No post hoc trajectory alignment is applied. Mean position error (MPE) is the time average of that distance; position root-mean-square error (RMSE) gives greater weight to large deviations. Equations (17)–(19) define position error, MPE, RMSE, and peak error. Trial metrics are summarized by means and sample standard deviations across all 60 trials. A high-error event is a maximal uninterrupted run above a fixed threshold lasting at least five frames. Equation (20) counts all frames in each qualifying run, including the first four. Event counts are averaged per trial and are not failure probabilities.

(17) $$e_{k}=\|{\hat{p}}_{k}-p_{k}^{\mathrm{ref}}{\|}_{2}$$

(18) $$\bar{e}=\frac{1}{N}\sum_{k=1}^{N}e_{k},\;\mathrm{RMSE}=\sqrt{\frac{1}{N}\sum_{k=1}^{N}e_{k}^{2}}$$

(19) $$e_{\mathrm{peak}}{=\max}_{1\leq \;k\leq \;N}e_{k}$$

(20) $$\mathrm{CHR}(h)=\frac{100}{N}\sum_{k=1}^{N}1[k\in E_{h,5}]$$

Here, $E_{h,5}$ is the union of maximal contiguous runs with $e_{k}>h$ and length at least five; h is 0.15, 0.30, or 0.50 m. Event frequency counts those runs. Recovery-associated event labels in diagnostic exports denote temporal association only, not proven causal recovery.

### 4.4. Position Accuracy in the Controlled Model

Table 3 summarizes all 60 corrected Monte Carlo trials. Ours-Full gives an MPE of 0.029 m, compared with 0.364 m for the controlled FAST-LIVO2-like variant. Using the baseline MPE as the denominator gives a reduction of approximately 92.0%. The between-trial standard deviation is 0.003 m and mean position RMSE is 0.034 m. The mean per-trial peak is 0.112 m across the same 60 trials, not the peak of a selected sequence. These results apply to known-map registration and synthetic visual poses. They do not quantify improvement over official FAST-LIVO2 or show how much benefit would remain with raw-sensor processing and an evolving estimated map.

**Figure 4:**
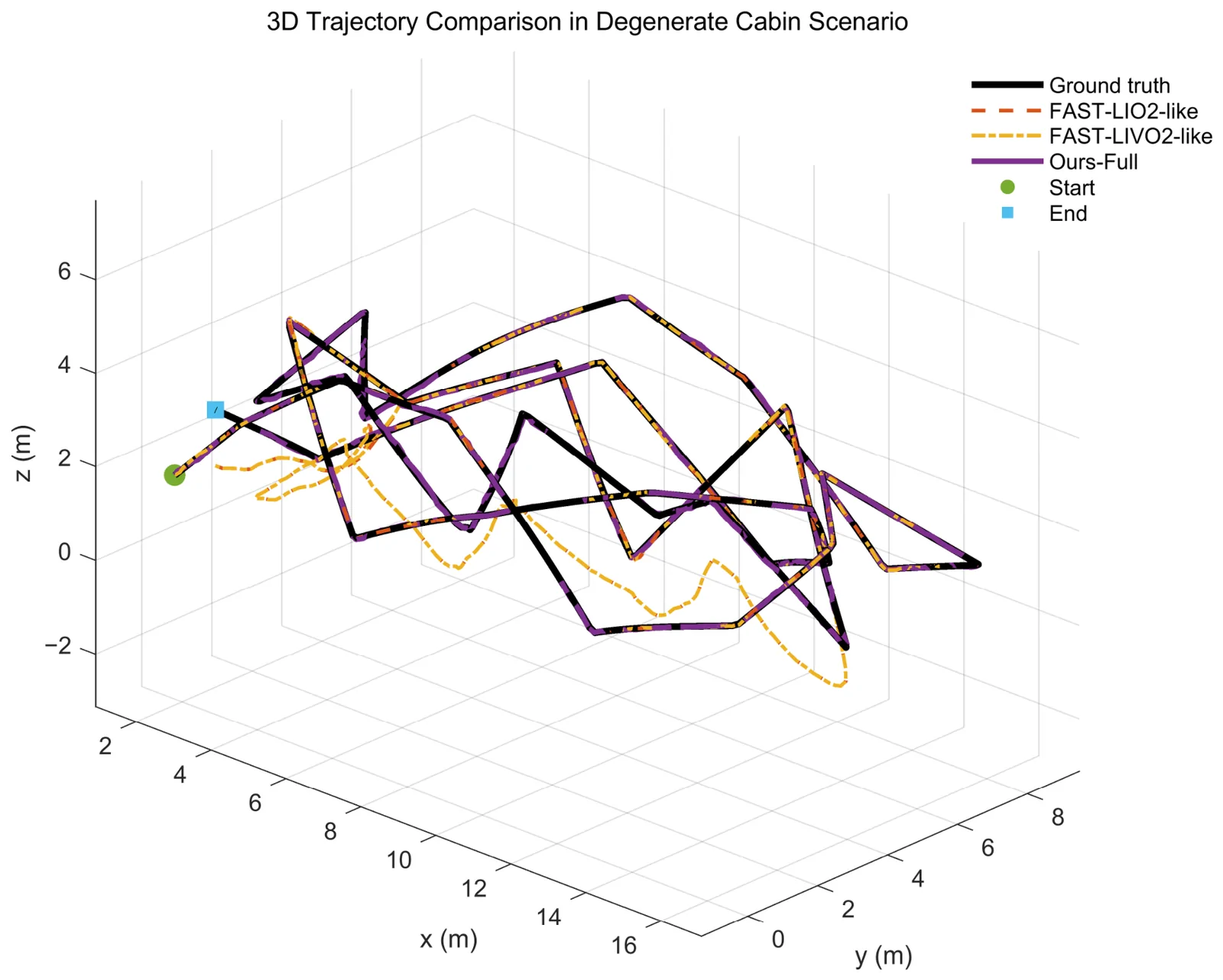
Representative position-and-yaw simulation trajectories. The two -like methods are controlled mechanism baselines, not official open-source executions. The synthetic reference is used for synthetic data generation and evaluation, not for filter correction, gating, or recovery. The displayed subset comprises the synthetic reference, FAST-LIO2-like, FAST-LIVO2-like, and Ours-Full; the other two variants are compared in Figure 5. The green circle and cyan square indicate the start and end, respectively. Nearly coincident trajectories overlap in this view.

Figure 4 shows one representative trajectory; Supplementary Figure S1 provides local projections. The plots show where the estimates differ in that sequence. Table 3, rather than this single example, supports the repeated-trial comparison.

**Figure 5:**
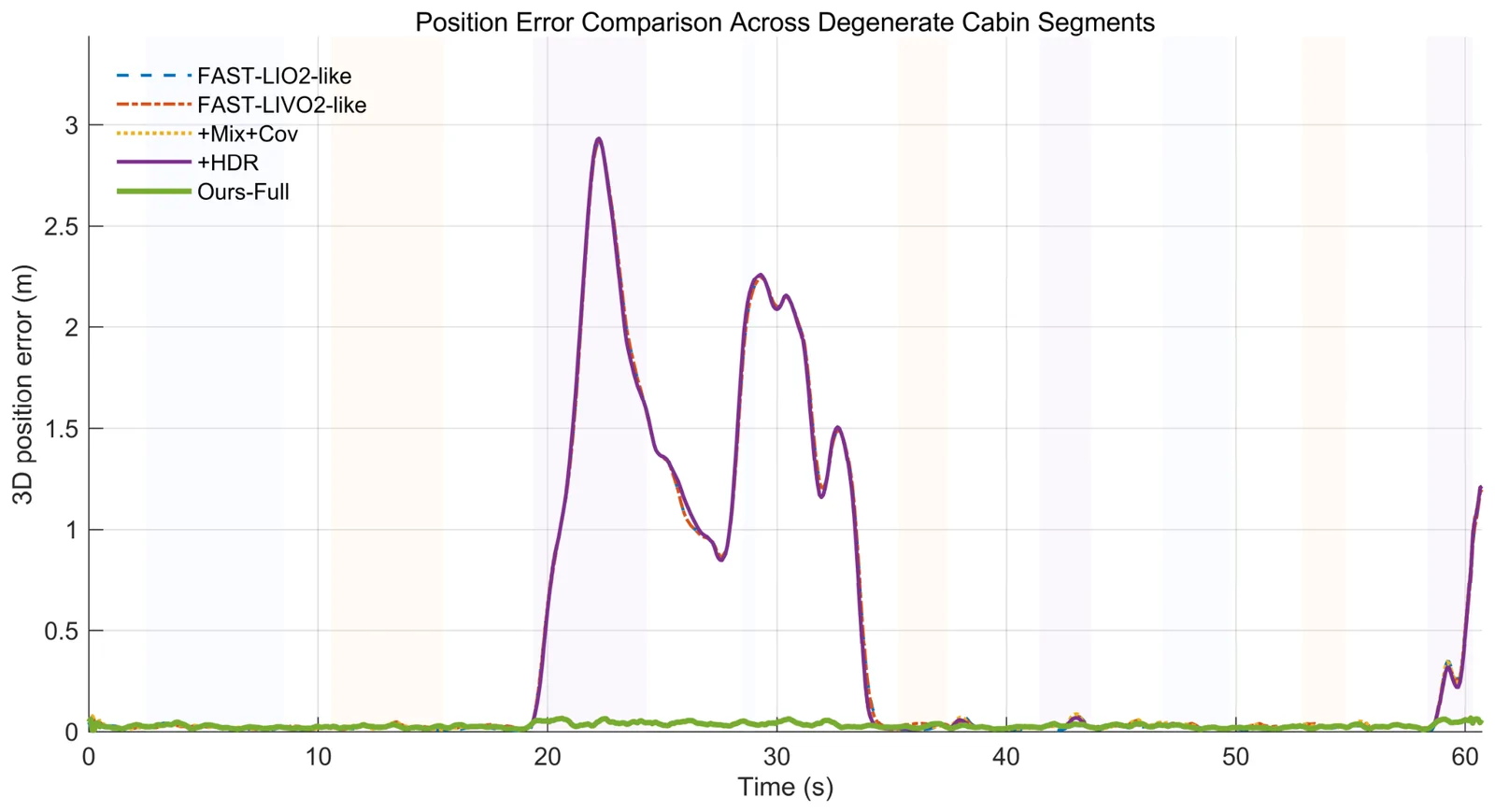
Framewise position errors for the five controlled simulation variants in one representative sequence. The shaded intervals indicate the prescribed synthetic degeneracy segments. Light blue, light orange, and light purple backgrounds denote prescribed geometric, photometric, and compound degeneracy intervals, respectively; unshaded intervals are nominal.

The four partial configurations have nearly overlapping errors in Figure 5, while Ours-Full has substantially lower error. Table 3 also shows little change from the LiDAR-only baseline through +HDR. The paired tests in Section 4.8.1 attribute most of the measured improvement to LiDAR innovation screening. They do not establish an independent mean-accuracy benefit from covariance adaptation or adaptive mixing. The HDR-equivalent proxy remains an assumed observation model, not a test of image-level compensation. These conclusions use the repeated trials, not only the plotted sequence.

### 4.5. Observation-Quality Response

**Figure 6:**
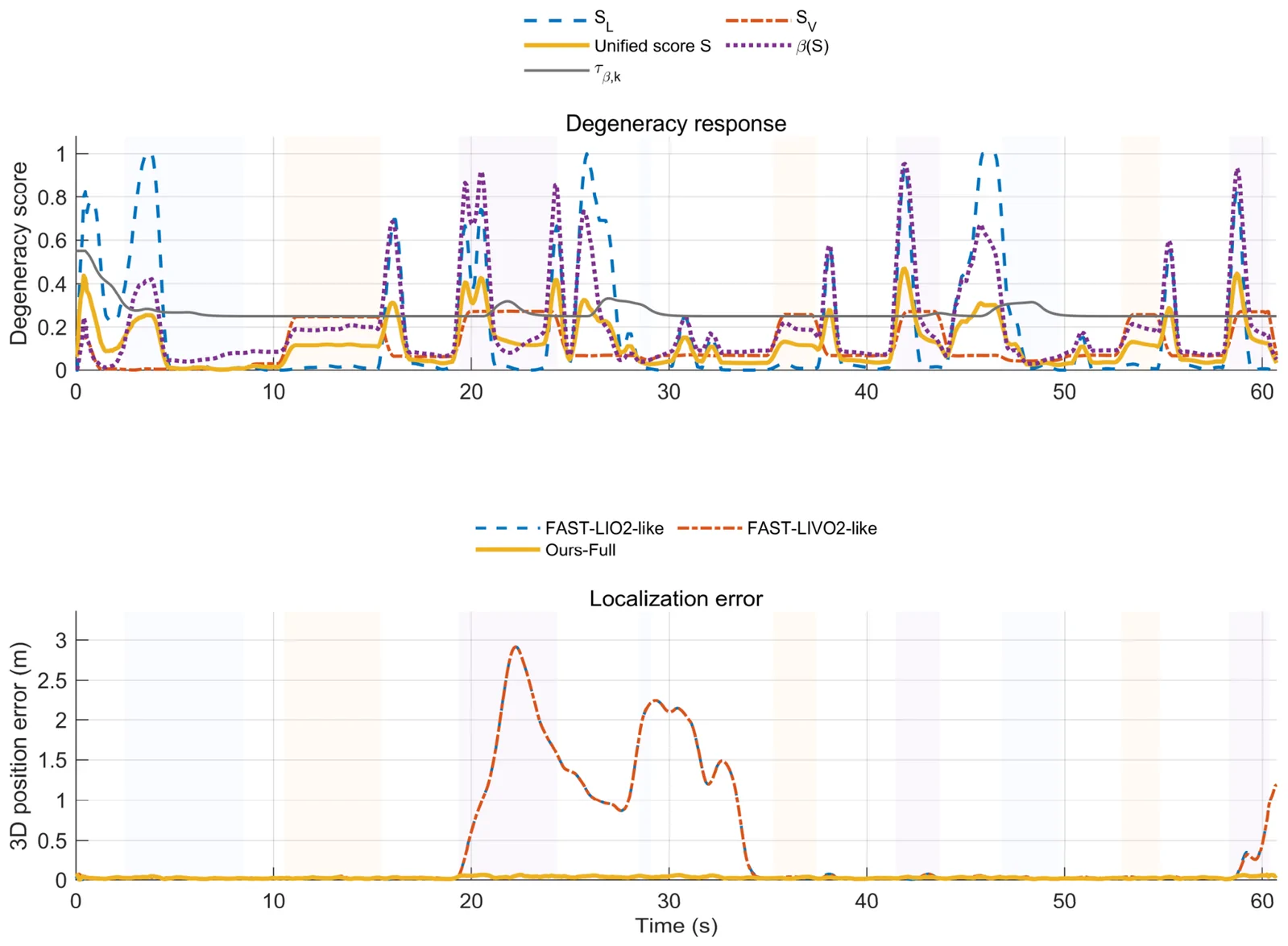
Geometric, visual, and unified quality indicators, nominal NDT mixing weight, and localization error in the representative synthetic sequence. Score-error association is diagnostic rather than a calibrated predictor of failure. Light blue, light orange, and light purple backgrounds denote prescribed geometric, photometric, and compound degeneracy intervals, respectively; unshaded intervals are nominal.

Figure 6 compares geometric and visual quality scores with position error. The scores use only current and past registration and image statistics. Reference error is added to the plot for diagnosis. A score peak need not coincide with an error peak: the score describes current observation quality, while drift builds over time. Keeping both channel scores beside the fused score helps identify degradation confined to one channel.

Supplementary Figure S2 shows the parameter response. Section 4.8.1 evaluates covariance and mixing changes while retaining screening and other safeguards; the paired-difference intervals include zero for these contrasts.

Supplementary Figure S3 distinguishes branch availability from update acceptance and rejection. Its innovation norm is $\sqrt{\mathrm{NIS}/4}$, computed using predicted state covariance and the pre-gate observation covariance. The plotted visual scale describes the complete pre-gate scale; the actually used covariance, including any gate or Huber inflation, is saved separately.

### 4.6. Repeated-Trial Robustness and Worst-Case Analysis

**Figure 7:**
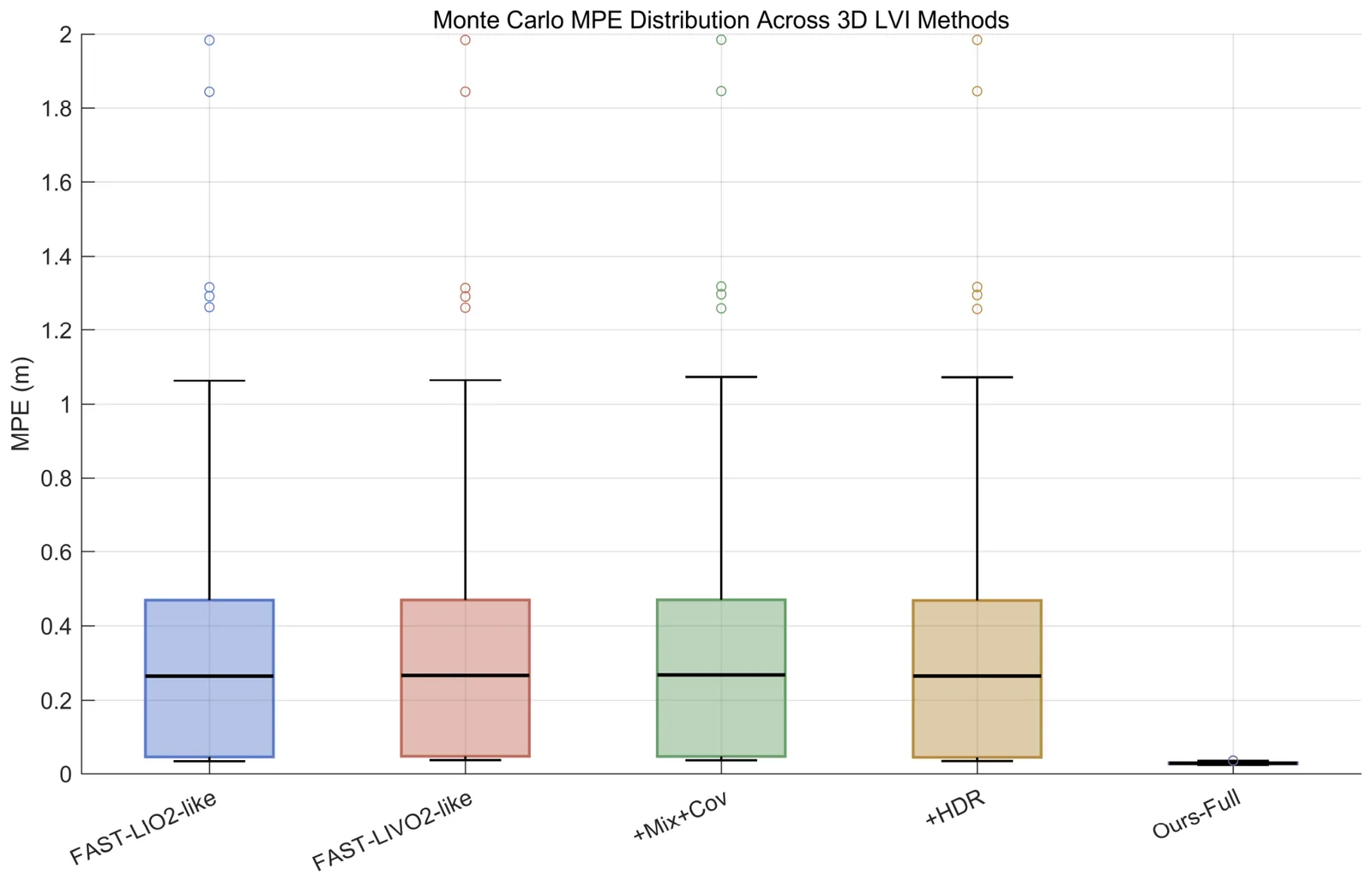
The distribution of trial-level mean position error (MPE) over all 60 controlled trials. Outliers remain part of the numerical aggregates. The -like labels denote simulation-level baselines. All five variants are included; the small Ours-Full box reflects its narrow interquartile spread at this vertical scale.

Figure 7 shows all 60 trial-level mean position errors. For Ours-Full, the median is 0.029 m, the interquartile range is 0.003 m, and the worst value is 0.036 m. The full distribution is retained, including extreme runs. Supplementary Figure S4 presents the corresponding means and between-trial standard deviations.

Table 4 reports consecutive high-error ratios using the unchanged thresholds. Ours-Full has mean ratios of 0.00%, 0.00%, and 0.00% at 0.15, 0.30, and 0.50 m. FAST-LIVO2-like has a ratio of 20.12% at 0.15 m. Supplementary Figure S5 shows the same results. A high-error event is not necessarily trajectory loss. Zero means that no run met the threshold and duration rule in these 60 controlled trials, not that error was absent.

**Figure 8:**
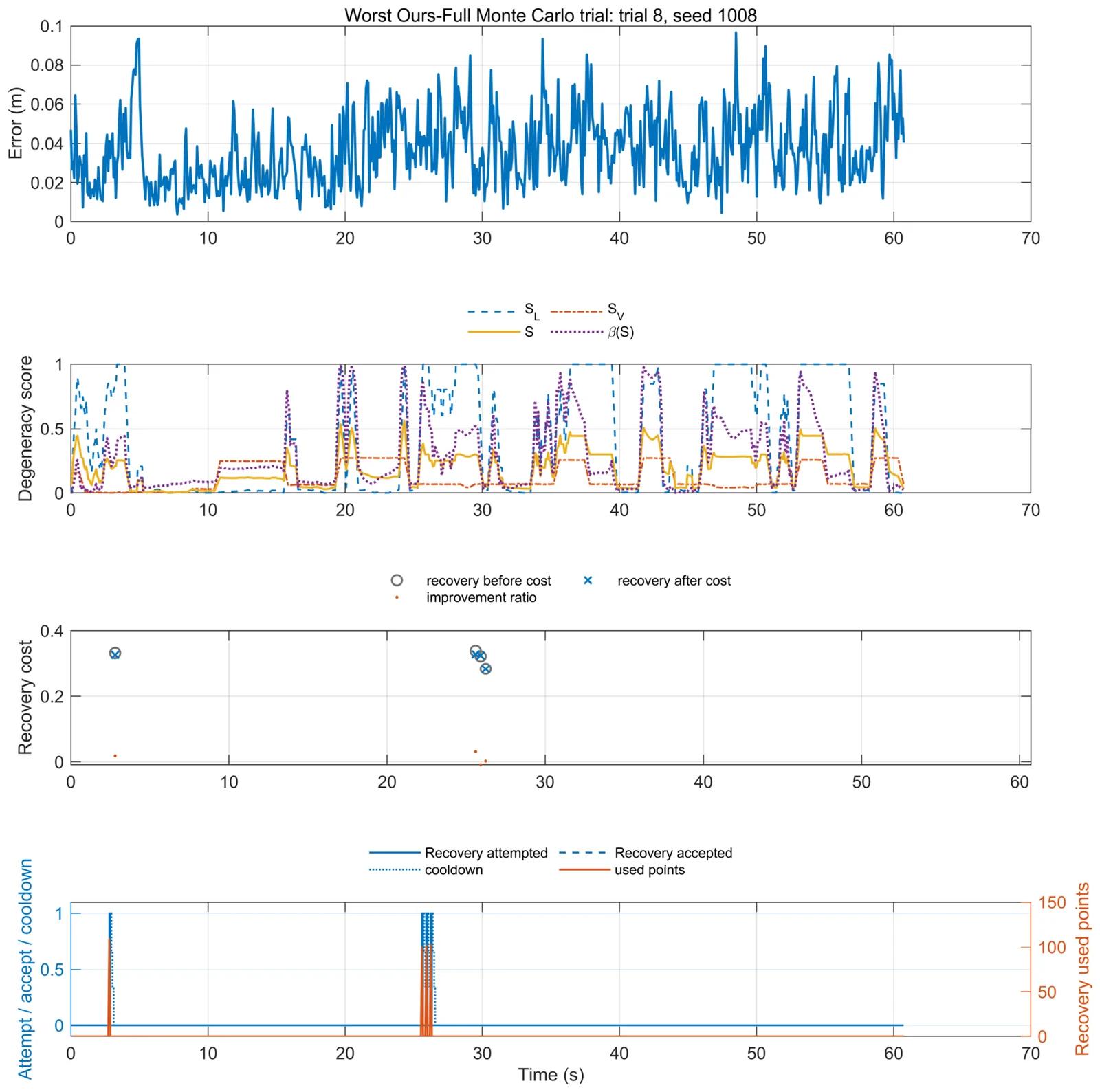
Worst corrected Monte Carlo trial by mean position error (trial 8, seed 1008). Reference error, quality scores, evaluated recovery costs, and actual trigger/acceptance states are shown. Missing cost samples denote unevaluated candidates, not zero cost.

Figure 8 diagnoses trial 8 (seed 1008), selected by the largest Ours-Full mean position error. Its mean error is 0.036 m, peak error is 0.096 m, and consecutive high-error ratio at 0.15 m is 0.00%. No recovery correction was accepted in this trial; paired recovery effects are evaluated in Section 4.8.7. The worst sequence remains part of every aggregate.

### 4.7. Computational Cost

**Figure 9:**
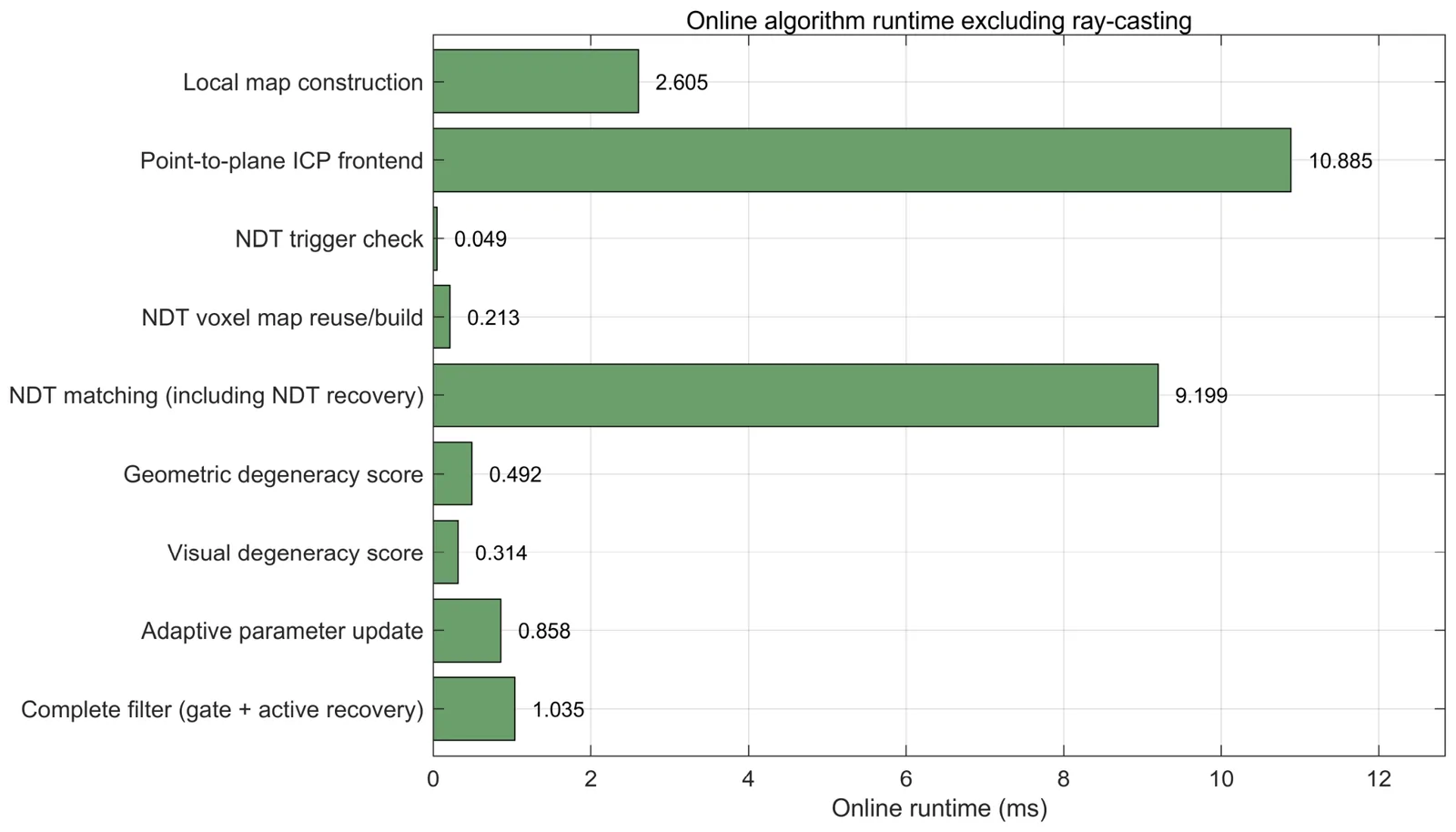
The non-overlapping runtime components of the reduced-order implementation. Ray-casting and synthetic image generation are excluded; the filter total already contains visual gating and active recovery.

Table 5 and Figure 9 report a representative processing time of 25.65 ms per frame for the reduced-order implementation. Its reciprocal is 39.0 Hz. Timers cover frames 2-760 and do not overlap; the complete filter timer already includes visual gating and active recovery. Synthetic generation takes 315.04 ms per frame and is excluded. Ray-casting is used only for synthetic LiDAR data generation and is excluded from online runtime evaluation. Raw-image alignment and estimated-map feedback are not implemented and are not timed. Ablation timings belong to their own replay protocol. The benchmark does not measure embedded-device latency or power consumption.

### 4.8. Internal Ablation and Parameter Sensitivity

Each internal ablation disables one configured component. The full configuration uses the same result and parameter record as the main experiment. Always-on NDT reruns registration on the stored scans and images, using the same velocity, yaw-rate, and visual pose observations. Changing registration sampling cannot change the later sensor-noise sequence. Table 6 includes all 60 paired seeds.

Table 6 and Supplementary Figure S7 show that the modules have different and sometimes unresolved roles. Disabling the visual gate changes MPE from 0.02920 m to 0.02921 m, which does not establish a meaningful accuracy gain from that gate. Removing map consistency gives 0.02899 m; the fixed reference map does not exercise the map-contamination mode that this safeguard is intended to prevent. Removing weak-direction damping raises the mean peak from 0.112 to 0.122 m, providing limited evidence for peak-error suppression in this protocol. Always-on NDT gives 0.02920 m at 34.50 ms per frame, compared with 20.91 ms for the full configuration, a 39.4% processing-time reduction with conditional activation. Recovery is accepted only once across 60 trials, and its removal changes MPE from 0.02920 m to 0.02919 m. This is insufficient to demonstrate recovery effectiveness. All six variants have no qualifying high-error runs at 0.15 m, so the present protocol cannot rank their sustained-error protection.

Section 4.8.1, Section 4.8.2, Section 4.8.3, Section 4.8.4, Section 4.8.5, Section 4.8.6 and Section 4.8.7 examine these outcomes through attribution, stress, and transfer tests. Full denotes the original complete configuration; Reduced is a separately specified comparison.

The supplementary tests ask which mechanisms explain the error gap and how the filter handles additional pose-observation anomalies. They retain all 60 original seeds. Default replay reproduces every archived full trajectory exactly, leaving the main experiment and figures unchanged. The supplement MATLAB Controlled Mechanism Validation provides Tables SM1 and SM2 and Figures SM1–SM4.

#### 4.8.1. Paired Mechanism Attribution

The attribution study replays the original observations for seeds 1001–1060. Independent switches disable Huber weighting, LiDAR innovation screening, or both while retaining the other safeguards. Additional variants disable the adaptive covariance group, fix mixing at 0.5, or combine those interventions. The covariance group includes adaptive process scaling, base-covariance feedback, and modality-specific inflation; visual confidence scaling and separate safeguards remain active. Invalid observations remain rejected when innovation screening is disabled.

Full has mean position error 0.029198 m. Removing LiDAR screening raises it to 0.349916 m; removing both screening and Huber weighting gives 0.359740 m. Removing Huber alone gives 0.029200 m. Disabling LiDAR screening produces a mean paired increase of 0.320718 m, with a 95% bootstrap interval of [0.219987, 0.438621] m. The effect of Huber weighting depends on whether screening is active. Supplementary Material S2, Table SM1 and Figure SM1 report all contrasts.

Mean error is 0.029187 m without adaptive covariance, 0.029179 m with fixed mixing, and 0.029230 m with both adaptations removed. All three paired-difference intervals include zero. The tests do not show an independent mean-accuracy gain from either rule. Other safeguards still use degeneracy information, so this is not a test of removing all quality information. Intervals use 5000 whole-trial bootstrap resamples, without adjustment for multiple comparisons. They describe seed variation in one simulated scene, not generalization to other environments.

A separate policy comparison uses a common archived always-on NDT observation pool. Adaptive mixing, fixed mixing at 0.5, ICP-only, and NDT-only give mean errors of 0.02920, 0.02946, 0.02915, and 0.03460 m, respectively (Supplementary Material S2, Table SM2 and Figure SM2). Single-source policies do not substitute the other registration source when invalid. These pose-observation results do not demonstrate universal superiority of adaptive mixing or compare independently executed registration objectives. Figure SM4 reports filter-only replay timing, excluding cached front-end computation, and does not replace the complete profile in Section 4.7.

#### 4.8.2. Additional Pose-Observation Corruption

The stress tests add the same 0.4 m translation in a seeded random direction and a 5-degree yaw disturbance to LiDAR and visual pose observations. Contiguous corruption starts at 20 s and lasts 6, 12, or 24 s; scattered corruption uses probabilities of 0.05, 0.10, or 0.20. Masks are nested within each study and shared across paired configurations. The original scene, observation noise, thresholds, and 60 seeds are preserved; reference errors do not select disturbances.

For Full, mean position error rises from 0.066 m at 6 s to 0.107 m at 12 s and 0.178 m at 24 s. Scattered corruption gives 0.02943, 0.02976, and 0.03076 m at the three probabilities. Supplementary Material S2, Figure SM3 reports mean errors and consecutive high-error ratios. Screening handles the tested scattered anomalies better than sustained corruption shared by both channels. The test does not establish bounded error during prolonged loss of reliable observations.

These tests change pose observations, not scans, images, Hessians, quality indicators, or maps. They do not simulate a newly generated planar scene, exposure loss, or sensor failure. Section 4.8.5 and Section 4.8.7 separately test visual screening and recovery; sensor-level degradation remains untested.

#### 4.8.3. Parameter Sensitivity

Supplementary Table S1 and Figure S8 vary one base parameter or threshold at a time on one fixed sequence, with causal adaptation retained. This is neither an independent parameter-selection study nor another Monte Carlo experiment. Adaptation can reduce the lasting influence of an initial setting. These results do not identify optimal coefficients or establish transfer to a new scene. A stronger selection protocol would use separate development sequences to set candidate ranges, freeze the chosen configuration, and evaluate it on held-out geometry and degradation conditions.

#### 4.8.4. Fixed Reduced Configuration

A reduced configuration was fixed before replay: adaptive covariance scaling, candidate-map protection and active recovery were disabled, and mixing was fixed at 0.5. LiDAR screening, Huber weighting, visual confidence and innovation screening, weak-direction damping, and the archived front-end inputs were retained. Across 60 paired trials, MPE was 0.029198 m for Full and 0.028923 m for Reduced. The paired difference (Reduced minus Full) was −0.000276 m, with a trial bootstrap 95% interval of [−0.000474, −0.000081] m. This is a joint simplification, not attribution to an individual component. No non-inferiority margin was specified, so equivalence is not claimed. Reduced had lower mean error in this protocol; the additional complexity of Full is not justified by mean accuracy on these inputs. Mean trial peaks were 0.11220 m for Full and 0.09321 m for Reduced; the largest individual peaks were 0.24187 and 0.11732 m, respectively. The comparison does not test prevention of feedback-map contamination or broader operating conditions.

Supplementary Material S3, Table ST1 and Figure ST1 in the targeted-validation supplement report accuracy and filter-only replay cost. This timing excludes registration, voxel construction, and simulation generation. It does not replace the complete processing profile in Section 4.7. The main results and model parameters remain unchanged.

#### 4.8.5. Visual Innovation Screening Under Isolated Corruption

The visual-specific comparison retains the original LiDAR observations, propagation inputs, quality indicators and scene. Only synthetic visual pose observations receive the pre-specified additional translation of 0.4 m in one fixed random direction per trial and yaw of five degrees. Corruption lasts 6, 12 or 24 s from 20 s, or uses nested random masks with probabilities of 0.05, 0.10 and 0.20. Each condition has 60 paired trials. The control disables only visual innovation rejection and its soft covariance inflation; confidence-based covariance scaling, low-confidence rejection and invalid-input rejection remain enabled. Innovation-dependent adaptive covariance evolution is also retained. Thus this is not a comparison with an entirely unprotected visual branch.

At corruption probabilities of 0.05, 0.10 and 0.20, Full produced MPE values of 0.02912, 0.02919, and 0.02946 m, compared with 0.03155, 0.03472, and 0.04486 m without the visual innovation gate. Under persistent corruption lasting 6, 12 and 24 s, the corresponding Full values were 0.05029, 0.07383, and 0.11681 m, versus 0.06308, 0.09984, and 0.17267 m without the gate. Supplementary Material S3, Tables ST2 and ST3 report rejection proportions and paired intervals. Rejection of an injected observation tests compatibility with the evolving prediction, not detection of its true cause. Sustained bias can remain compatible as the estimate and working covariance evolve. These results concern a pose proxy, not pixel-level HDR alignment.

Screening lowers aggregate error in these added conditions, but Full still reaches a mean consecutive high-error ratio of 23.96% at 24 s. Without the gate, MPE is lower in 15 of the 60 trials at that duration. Screening helps on average here; it does not help every trial or remove all sustained biased updates.

Figures ST2 and ST3 distinguish scattered and persistent corruption and show rejection on injected and uninjected frames. Uninjected frames retain the original noise and anomalies. Their rejection proportion is not a known false-positive rate. The paired whole-trial bootstrap intervals are descriptive and unadjusted for multiple comparisons. These 60 seeds share one scene and cannot establish cross-environment transfer.

#### 4.8.6. Frozen-Parameter Transfer Tests

Two conditions were fixed before execution: reverse traversal in the original cabin, and a 22 by 6.4 by 5.2 m cabin with the normalized route generator. Each uses 60 new paired seeds and all 760 samples. Scans and registration are regenerated with unchanged noise and estimator parameters; Full and the fixed Reduced configuration share identical inputs.

Reverse traversal gives MPE of 0.029198 m for Full and 0.029413 m for Reduced. The paired difference (Reduced minus Full) is 0.000214 m, with a 95% whole-trial bootstrap interval [0.000052, 0.000379] m. Full has lower mean error in this condition.

The resized cabin gives MPE of 0.029935 m for Full and 0.029207 m for Reduced. The paired difference (Reduced minus Full) is −0.000728 m, with a 95% whole-trial bootstrap interval [−0.000892, −0.000573] m. Reduced has lower mean error in this condition.

The mean-error differences are below 1 mm and favor different configurations in the two conditions. Table SV1 and Figure SV1 report the conditions separately. These tests neither establish broad generalization nor separate geometry effects from trajectory effects. No configuration is selected or retuned using these results.

#### 4.8.7. Paired Recovery Assessment

Section SV2 of Supplementary Material S4 (Transfer and Recovery Validation) compares recovery enabled and disabled on the 60 original trials and on matched 6, 12 and 24 s pose disturbances. Only the active-recovery switch changes. Disturbances affect either LiDAR poses alone or both observation channels; scans, map, prediction inputs, four-attempt budget and acceptance thresholds remain fixed.

LiDAR-only corruption gives enabled/disabled MPE of 6 s: 0.02964/0.02963 m; 12 s: 0.03023/0.03022 m; and 24 s: 0.03391/0.03390 m.

Common pose corruption gives enabled/disabled MPE of 6 s: 0.06617/0.06626 m; 12 s: 0.10650/0.10648 m; and 24 s: 0.17811/0.17813 m.

Across 420 trial-condition executions, recovery makes 1397 attempts and accepts nine corrections. Of these, six increase the immediate reference position error. The largest accepted translation is 0.17135 m. Repeated conditions are not independent trajectory samples. Supplementary Material S4, Tables SV2–SV4 report paired intervals, high-error duration, and settling, with a defined censoring rule.

For disabled-minus-enabled MPE, one of the six corruption-condition intervals lies above zero, three lie below zero, and two include zero. All absolute mean-error differences are below 0.1 mm. Together with the acceptance statistics, these results show small, mixed recovery effects rather than a reliable improvement. Recovery therefore remains an optional experimental component.

Because scans and the known map remain unchanged, the test covers pose-observation corruption only. Recovery from corrupted maps or raw-sensor failures remains unvalidated. Timings from parallel execution do not replace the main runtime measurements.

### 4.9. Separate Field Point-Cloud Representation Study

Photographs and point clouds were collected on site in a steel-plate compartment mock-up with repeated panels, local reflections, auxiliary lighting, and railing occlusions. This study examines how the measured geometry is represented, not how accurately the algorithm localizes. Synchronized estimator logs and an independent trajectory reference are unavailable. The files cannot establish real-data absolute trajectory error (ATE), relative pose error (RPE), localization robustness, or complete-pipeline execution.

**Figure 10:**
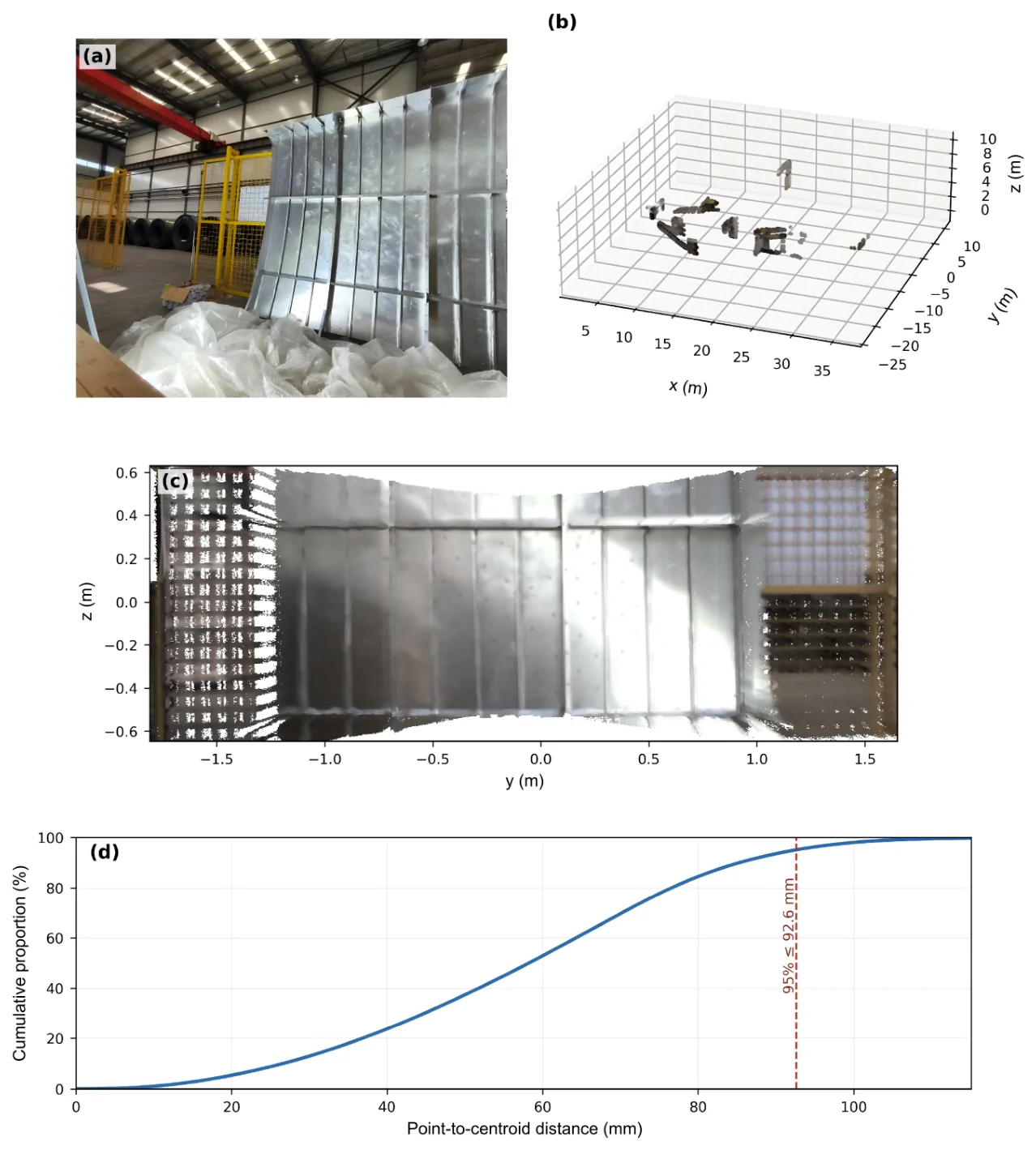
Field-measured compartment data and voxel representation: (**a**) steel-plate mock-up; (**b**) 0.15 m voxel centroids; (**c**) orthographic color projection; and (**d**) raw-point-to-centroid distance distribution.

Figure 10 shows the acquisition setting and voxel representation. We measure the reduction in stored points and the distance from each raw point to its voxel centroid. Neither measure is a trajectory error.

The main point-cloud file contains 13,690,771 finite points. Voxelization at 0.15 m gives 3435 occupied-voxel centroids, matching the supplied reduced cloud. The point count falls by 99.9749%, or approximately 3985.7:1. One centroid is retained for each occupied voxel. This describes compression at the chosen resolution, not preservation of every geometric detail or localization constraint.

**Figure 11:**
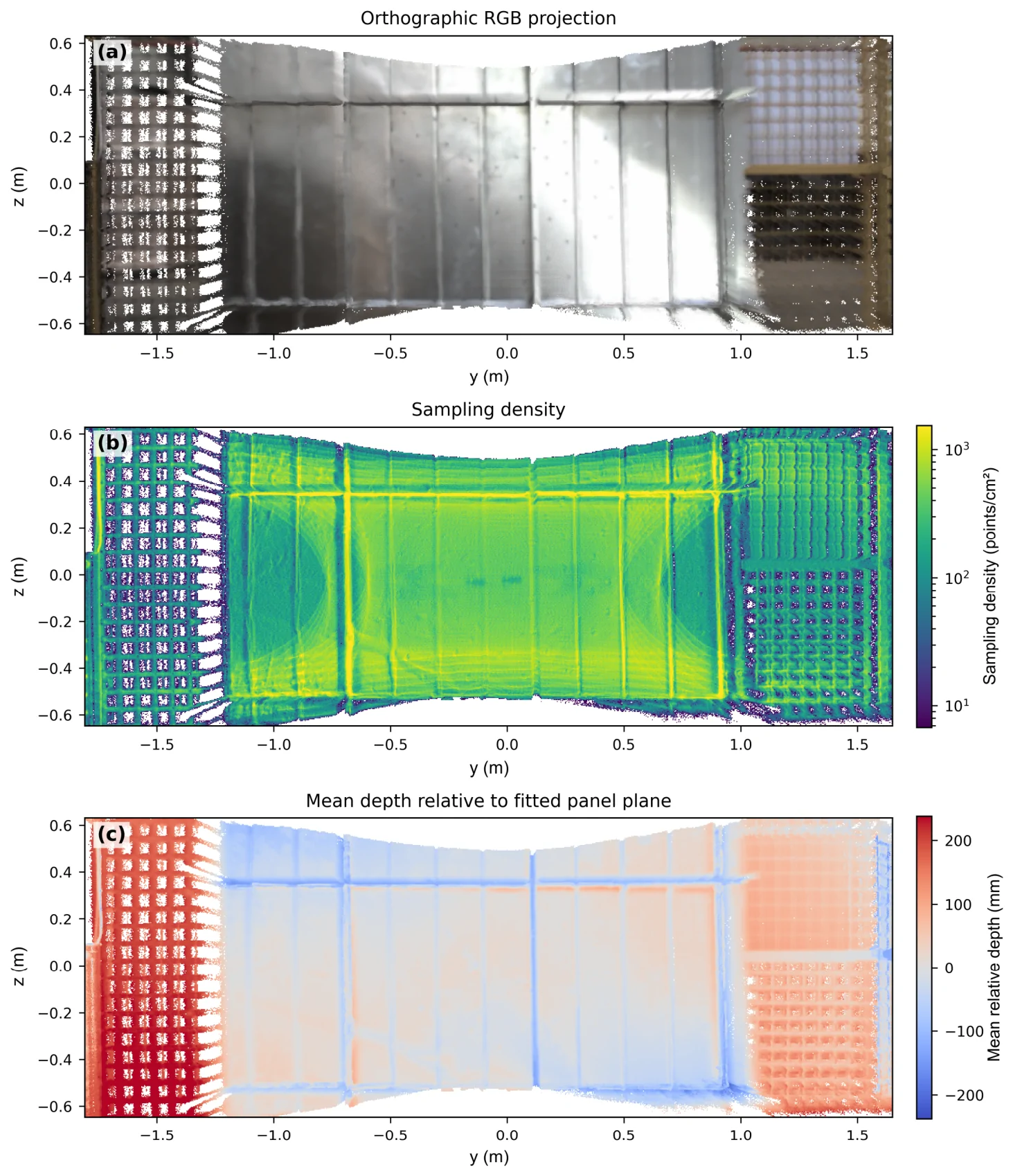
The local attributes of the field-measured principal region: (**a**) orthographic color projection; (**b**) sampling density; and (**c**) mean depth relative to the fitted principal steel-plate plane.

The recomputed median and 95th-percentile point-to-centroid distances are 58.5 and 92.6 mm. They measure the geometric approximation introduced by voxelization. Figure 11 shows the main measured region and its sampling pattern in more detail.

Figure 11 shows an approximately 3.46 m by 1.28 m projected region with 86.96% grid coverage. These descriptors depend on the projection; they are not independent measurements of area or completeness. Sampling density falls near openings, boundaries, and occlusions. Relative depth contains both structural relief and registration effects, so it should not be read as steel-plate flatness.

The field study quantifies the storage reduction and geometric approximation of the site-acquired point cloud at the selected voxel resolution.

### 4.10. Measurement Interpretation and Limitations

The evidence supports different roles for different components. LiDAR innovation screening accounts for the dominant measured accuracy gain, and conditional NDT reduces computation in the paired timing comparison. Visual screening helps on average under the added visual-pose corruption, not uniformly across trials. Independent mean-accuracy gains from mixing and covariance adaptation remain unestablished; map-contamination prevention is untested with a fixed registration map. Active recovery has small, mixed effects and remains optional. Raw-image alignment, raw-IMU mechanization, estimated-map feedback, and independently referenced real trajectories are outside this evaluation.

#### 4.10.1. Full Motion and Inertial Bias Estimation

A full inertial extension would estimate position, velocity, orientation, and gyroscope and accelerometer biases. With calibrated gravity and sensor extrinsics held fixed, a conventional local error state has 15 components: three each for position, velocity, attitude error, and the two bias vectors. This is larger than the six degrees of freedom of a pose. Raw angular-rate and specific-force measurements would replace velocity and yaw-rate inputs. The implementation would need bias-aware propagation, scan deskewing, and consistent measurement Jacobians. Estimating gravity or extrinsics online would add further states.

Degeneracy detection would then examine translational and rotational constraints using a scaled six-dimensional pose information matrix. Weak scan geometry and poor temporal observability of inertial biases are different limitations: a favorable registration spectrum does not establish bias observability. Bias-state cross-covariances must remain in propagation and measurement updates, and observation inflation must not replace an inertial process-noise model. Innovation thresholds would be chosen for the actual measurement dimension and assessed on independent sequences, rather than copied from the current four-dimensional gate. Larger covariance operations, raw-image alignment, deskewing, and map maintenance would add cost. Conditional NDT and voxel reuse may limit registration work, but their net benefit and total latency require profiling the full implementation; the present 25.65 ms value does not quantify that extension.

#### 4.10.2. Degradation and Fault-Aware Navigation

Related integrated-navigation studies address reliability at a different level. Xu et al. [26] combine redundant strapdown inertial navigation system (SINS) fault diagnosis with initial alignment under large misalignment angles. Their use of sensor redundancy to identify gyroscope bias faults differs from rejecting an inconsistent pose observation. Xu et al. [27] use inertial aiding within star-sensor processing to retain attitude solutions when stellar observations are scarce. This illustrates the value of retaining informative partial measurements, but its sensor model and attitude-availability results cannot be transferred directly to confined-compartment LVI.

Validity and innovation checks let one observation channel correct the prediction when the other degrades. They do not identify whether disagreement comes from hardware failure, poor geometry, incorrect correspondences, or an inaccurate prediction. The model does not diagnose persistent propagation-input bias, and losing both observation channels can still cause drift. A sensor-level system would need health monitoring, fault isolation over time, and a controlled return to using recovered channels. Camera outages, LiDAR overlap loss, inertial bias faults, and joint channel loss require separate tests. They are not demonstrated capabilities of the current model.

#### 4.10.3. Conditional Computation and Prospective Collaboration

Conditional NDT and reused voxel statistics reduce local registration work. Federated learning-based distributed control [28] offers a different approach to sharing model information across robots. The present estimator does not use distributed computation or learning.

A multi-robot extension could share quality summaries, validity flags, or selected map statistics instead of raw scans and images. It would need compatible score definitions, coordinate frames, and timestamps. Correlated estimates, communication delays, and unreliable updates would also need testing. The current rule-based policy has no learned parameters to aggregate.

#### 4.10.4. Sensor-Level Validation Pathway

The approximately 92.0% MPE reduction is a comparison between controlled mechanism variants, not official LVI systems. Testing a sensor-level implementation would also expose calibration error, motion distortion, tracking loss, and map feedback. The current protocol does not include these effects.

A practical next test needs synchronized raw LiDAR, camera, and IMU data; documented calibration; and an independent trajectory reference. A short mock-up recording with motion capture would be one option. Reference accuracy and availability should be reported. Reference measurements must remain outside the estimator updates.

The original and enhanced versions of a compatible official estimator, such as FAST-LIVO2 [10], should run on the same data, calibration, hardware, and frozen parameters. The recordings should include planar, repeated, poorly lit, low-overlap, and compound-degradation segments. Innovation, gating, NDT-activity, and recovery-cost logs would help distinguish front-end effects from fusion-policy effects.

Future recovery studies should evaluate event-based attempt budgets and candidate-acceptance checks based on correspondence support, overlap, and prediction compatibility. Parameters should be fixed on separate development inputs before paired recovery-on/off evaluation, with harmful accepts and sustained high-error intervals retained in the analysis.

Independently referenced sensor-level trajectories are the priority for follow-up work. Evaluation should retain incomplete runs and report position and orientation errors, alignment conventions, high-error intervals, and recovery duration. Covariance calibration requires separate assessment. End-to-end timing should include deskewing, visual alignment, and map updates, with mean and tail latency reported.

## 5. Conclusions

The controlled position-and-yaw study identifies LiDAR innovation screening as the dominant measured accuracy contributor and conditional NDT as a means of reducing computation. Visual screening provides condition-dependent protection under added pose-proxy corruption. The fixed Reduced configuration remains competitive, while transfer tests favor different configurations and recovery has small, mixed effects. The findings support selective use of the evaluated components rather than a uniform benefit from added complexity.

Across the 60 original trials, mean position error is 0.029 m with a between-trial standard deviation of 0.003 m. The consecutive high-error ratio at 0.15 m is 0.00%. Representative processing time is 25.65 ms per frame, excluding synthetic generation. These values describe the original known-map experiment with synthetic visual poses. Under sustained common pose corruption, mean error rises from 0.066 m at 6 s to 0.178 m at 24 s.

The field point-cloud study evaluates representation separately. The known-map and synthetic visual protocol leaves pixel-level alignment and estimated-map feedback untested. Official-system comparisons and synchronized sensor experiments with independent trajectory references are needed to assess practical LVI performance.

## Figures and Tables

**Table 1:** Main implementation parameters. Adaptive defaults are starting values, not constant per-frame settings.

Parameter	Default or Range	Role
α	0.55 initially; 0.25–0.75	Causal fusion weight; smoothing 0.85
$k_{\beta }$ $;\;\tau_{\beta }$	10; 0.55 initially	Sigmoid slope 6–18; threshold 0.25–0.75
$k_{L}$ $;\;k_{V}$	2.6; 2.3 base	Modality-specific covariance gains
$\gamma_{L}$ $;\;\gamma_{V}$	2.0 base; 1.3–2.3	Adaptive covariance exponents
NIS gates	9.4877 soft; 13.2767 hard	Four-dimensional, fixed before evaluation
Visual confidence	0.45 soft; 0.18 reject	Confidence weighting and rejection
Overlap	0.25 warning; 0.23 update	Map-quality protection and candidate admission
Keyframe novelty	0.45 m; 7 degrees	Translation/yaw thresholds; view/structure alternatives
Recovery score	0.60 for 2 frames	Cooldown 8/4 frames after acceptance/rejection; 4 attempts
Recovery acceptance	Cost < 0.99; more than 40 points	Overlap nondecreasing; visual ratio at most 0.995
Recovery increment	0.18 m; 2.5 degrees	45% small-step proposal, then bounded
Weak-direction protection	Relative eigenvalue 10^−3^	Damping 0.15–1; rotation length 1 m
Conditional NDT	Geometric score 0.42/0.28	Immediate activation; 3-frame hold including trigger frame (Table S5)
NDT input/iterations	220/460/650 points; 2/4/6	Nominal/high-degeneracy/recovery budgets
NDT voxel cache	0.70 m cells; 0.90 m shift	0.25 m scan sampling; yaw rebuild 12 degrees
Evaluation thresholds	0.15, 0.30, 0.50 m	Evaluation only, never estimator triggers
Consecutive frame count	5	All frames in qualifying contiguous runs counted

**Table 2:** Controlled simulation settings and reported quantities.

Item	Setting
Simulation platform	MATLAB R2020a; Intel Core i7-12700KF desktop, 32 GB RAM; four-dimensional model
Scene	Known synthetic metal compartment with stiffeners, pipes, reflective and degenerate regions
Trajectory	760 frames; 0.08 s interval; complex 3-D position-and-yaw trajectory
LiDAR observations	Ray-cast scans; known-map ICP/NDT poses; generation excluded from processing runtime
Visual observations	Synthetic image diagnostics and raw/HDR-equivalent four-dimensional pose proxies
Monte Carlo	60 trials; seeds 1001–1060; all trials retained
Compared methods	Five controlled mechanism baselines, not official public-code reproductions
Primary metrics	Time-mean position error, position RMSE, peak error, consecutive high-error ratio, processing time

**Table 3:** Position-error metrics over 60 controlled trials using mechanism baselines, not official FAST-LIO2 or FAST-LIVO2 implementations. MPE is the time-mean position error; standard deviations are across trials and peak errors are averaged per trial.

Method	Mean Error (m)	Std. (m)	Mean Peak (m)	High-Error (%)	Mean Events
FAST-LIO2-like	0.362	0.435	2.167	20.46	2.12
FAST-LIVO2-like	0.364	0.434	2.166	20.12	1.97
+Mix+Cov	0.364	0.435	2.191	19.92	2.47
+HDR	0.361	0.436	2.191	19.57	2.32
Ours-Full	0.029	0.003	0.112	0.00	0.00

**Table 4:** Sustained high-error statistics under the stated event definition.

Method	>0.15 m (%)	>0.30 m (%)	>0.50 m (%)	Mean Events at 0.15 m
FAST-LIO2-like	20.46	18.74	17.53	2.12
FAST-LIVO2-like	20.12	18.50	17.50	1.97
+Mix+Cov	19.92	18.39	17.53	2.47
+HDR	19.57	18.33	17.51	2.32
Ours-Full	0.00	0.00	0.00	0.00

**Table 5:** The representative-sequence processing profile of the reduced-order implementation. Online algorithm and synthetic data-generation components are reported separately.

Metric	Scope	Mean (ms)	Note
Complete processing	Reduced-order implementation	25.651	Disjoint timer sum
NDT trigger check	Processing	0.049	Hysteresis check
NDT voxel reuse/build	Processing	0.213	Map statistics
Synthetic generation	Simulation only	315.039	Scan and image generation excluded

**Table 6:** Internal ablation on 60 paired controlled trials. Each variant disables one named component; the full configuration is identical to that in the main comparison.

Variant	Disabled Module	Mean Error (m)	Peak Error (m)	High-Error (%)	Time (ms)
Without visual gate	Visual gate	0.029 ± 0.003	0.109 ± 0.013	0.00	21.07
Without map consistency	Map consistency	0.029 ± 0.003	0.111 ± 0.016	0.00	20.67
Without active recovery	Active recovery	0.029 ± 0.003	0.112 ± 0.021	0.00	20.79
Without weak-direction damping	Weak-direction damping	0.030 ± 0.003	0.122 ± 0.027	0.00	20.88
Without adaptive NDT trigger	Conditional NDT (always on)	0.029 ± 0.003	0.107 ± 0.018	0.00	34.50
Ours-Full	None	0.029 ± 0.003	0.112 ± 0.021	0.00	20.91

## Data Availability

Summary results have been included in this article and its Supplementary Materials; the raw simulation records and point cloud data from field measurements are not made publicly available.

## Supplementary Materials

The following supporting information can be downloaded at: https://www.mdpi.com/article/10.3390/s26216873/s1, Supplementary Material S1: Diagnostics and Implementation (Tables S1–S5 and Figures S1–S8); Supplementary Material S2: Controlled Mechanism Validation (Tables SM1 and SM2 and Figures SM1–SM4); Supplementary Material S3: Targeted Validation (Tables ST1–ST3 and Figures ST1–ST3); Supplementary Material S4: Transfer and Recovery Validation (Tables SV1–SV4 and Figures SV1–SV3, including Section SV2).

## Appendix A. Prospective Pixel-Level Visual Extension

This appendix describes a possible pixel-level extension. None of its equations is evaluated in the experiments. Implementation would require calibrated projection, depth and visibility checks, radiometric parameter estimation, and image-level optimization. Section 3.3 describes the visual proxy used in this study.

### Appendix A.1. Radiometric and Projection Models

Let a reference pixel u with known depth define a map point X. The warp w maps that point into the current image using the pose and camera intrinsic matrix $K_{\mathrm{cam}}$, as shown in Equation (A1).

(A1) $$w(u)=\pi (K_{\mathrm{cam}}(RX+t))$$

The map and camera coordinate conventions must be fixed before applying this projection. The subscript on $K_{\mathrm{cam}}$ distinguishes camera intrinsics from the Kalman gain K. A depth value and valid visibility mask are required to construct the correspondence.

The local affine radiometric model in Equation (A2) relates intensities at corresponding pixels.

(A2) $$I_{t}(w(u))\;\approx a_{t}I_{r}(u)+b_{t}$$

The current intensity is compared at the warped pixel, while the reference intensity remains at u. The scale $a_{t}$ and offset $b_{t}$ compensate first-order frame-to-frame exposure and brightness variations.

### Appendix A.2. Candidate Photometric and Gradient Residuals

The proposed photometric residual in Equation (A3) is normalized by an intensity-noise scale.

(A3) $$r_{\mathrm{photo}}=\frac{I_{t}(w(u))\;-a_{t}I_{r}(u)\;-b_{t}}{\sigma_{I}}$$

Image gradients must be compared in the same coordinate system. Equation (A4) uses the warp Jacobian to express the current-image gradient in reference-image coordinates and defines its normalization.

(A4) $$g(a)=\frac{a}{\|a{\|}_{2}+\varepsilon },\;g_{t}^{w}=J_{w}^{⊤}\nabla \;I_{t}(w(u))$$

Using this convention, Equation (A5) defines the gradient residual.

(A5) $$r_{\mathrm{grad}}=g(g_{t}^{w})\;-\;g(\nabla \;I_{r}(u))$$

Gradient normalization reduces sensitivity to global intensity scaling. The validity mask excludes weak-gradient and saturated pixels. Both the noise-normalized photometric residual and the gradient-direction difference are dimensionless. Equation (A6) combines them in the proposed objective.

(A6) $$L_{V}=\sum_{u\in \Omega }\rho_{p}(r_{\mathrm{photo}}^{2})+\lambda \sum_{u\in \Omega }\rho_{g}(\|r_{\mathrm{grad}}{\|}_{2}^{2})$$

Here, the valid-pixel set excludes occluded, out-of-frame, and saturated correspondences. The robust losses act on squared dimensionless residuals; λ controls their relative influence. Weak-gradient samples require rejection or separate confidence weighting. The combined objective is intended to retain complementary image information; that benefit has not been measured in this study.

In these proposed equations, Omega is the valid-pixel mask, *ρ_p_* and *ρ_g_* are robust losses, and lambda sets their relative weight. *σ_I_* in Equation (A3) is an intensity-noise scale; epsilon in Equation (A4) prevents division by zero. *J_w_* is the image-warp Jacobian, and g is the normalized gradient operator. These quantities would need implementation-specific definitions and calibration before sensor-level evaluation.

### Appendix A.3. Prospective Joint Objective

A sensor-level optimizer would linearize pixel residuals with respect to pose, radiometric scale, and offset. Equation (A7) shows only the pose subproblem. Radiometric variables and their Jacobians would need to be included or eliminated consistently. The current pose-proxy filter does not execute this objective.

The candidate linearized posterior objective in Equation (A7) contains the predicted state prior, LiDAR terms, and visual terms. It is not the objective executed in the reported pose-proxy experiments.

(A7) $$\min_{\delta x}\|\delta x{\|}_{{\left(P^{-}\right)}^{-1}}^{2}+\|r_{L}-J_{L}\delta x{\|}_{R_{L}^{-1}}^{2}+\|r_{V}-J_{V}\delta x{\|}_{R_{V}^{-1}}^{2}$$

In Equation (A7), delta x is the local pose increment, J_L and J_V are measurement Jacobians, and each weighted squared norm uses the indicated inverse covariance. Visual terms would need valid-pixel residuals and an appropriate noise model. The current experiment instead uses sequential pose corrections and the effective-gain Joseph update in Section 3.2. The image solver, its initialization, and its uncertainty mapping remain to be implemented and tested.
