# Supplementary material for: A Controlled Simulation Study of Degeneracy-Aware Pose Fusion in Confined Metal Compartments
Source: Sensors (Basel). 2026 Oct 30;26(21):6873. doi: 10.3390/s26216873 (PMC13766205; doi:10.3390/s26216873)
Supplement: Supplementary file 1 [file sensors-26-06873-s001.zip › Supplementary Material S1. Diagnostics_and_Implementation.pdf]

Supplementary Material

A Controlled Simulation Study of Degeneracy-Aware Pose Fusion in Confined Metal Compartments

Supplementary Figure S1

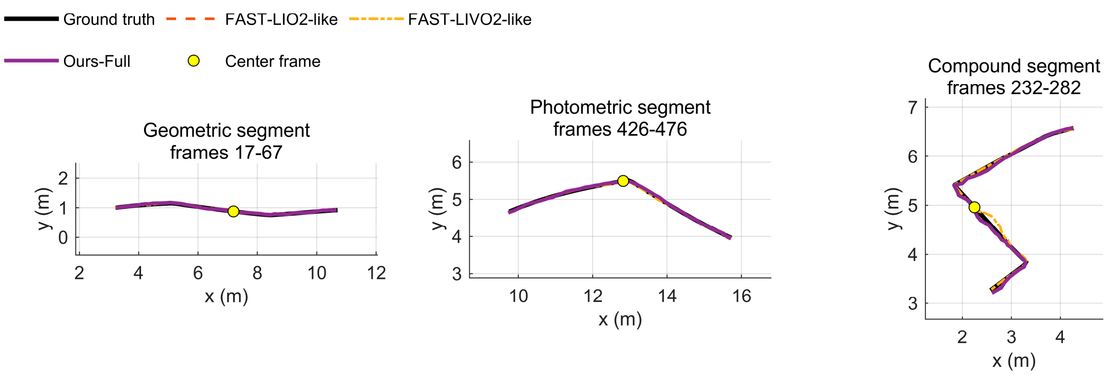

Figure S1. Local projections of the representative controlled trajectory.

Supplementary Figure S2

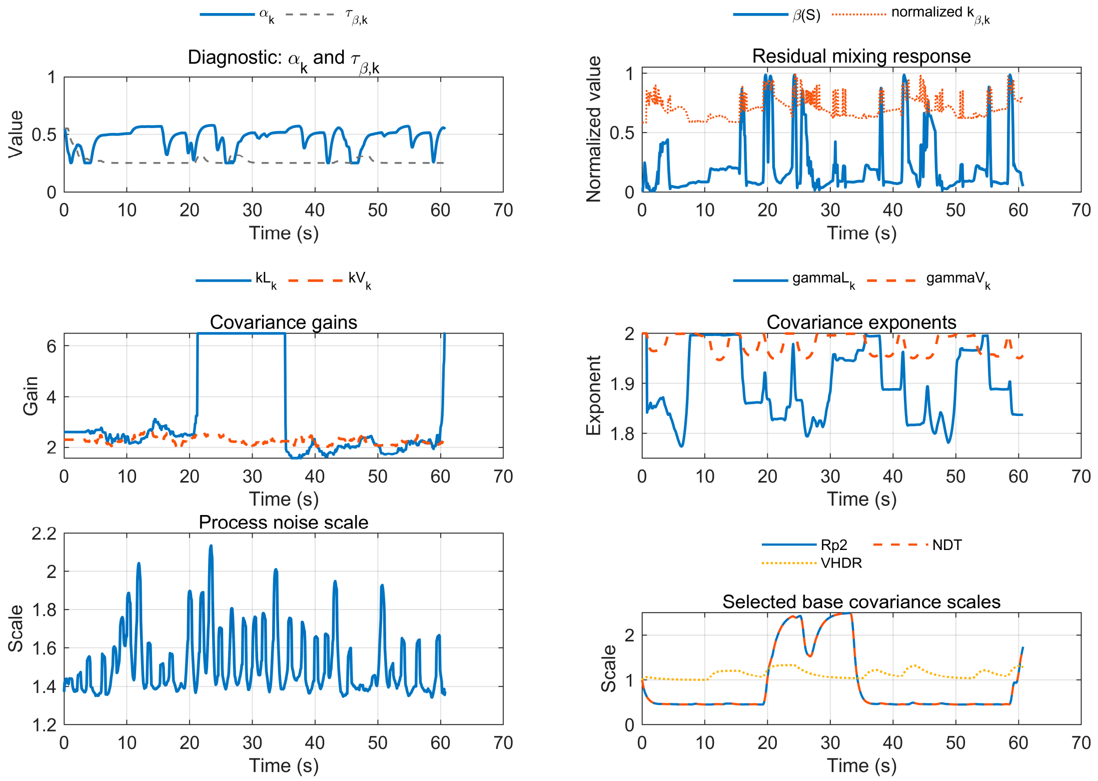

Figure S2. Causal parameter traces in the representative sequence; controller activity is not proof of improvement.

Supplementary Figure S3

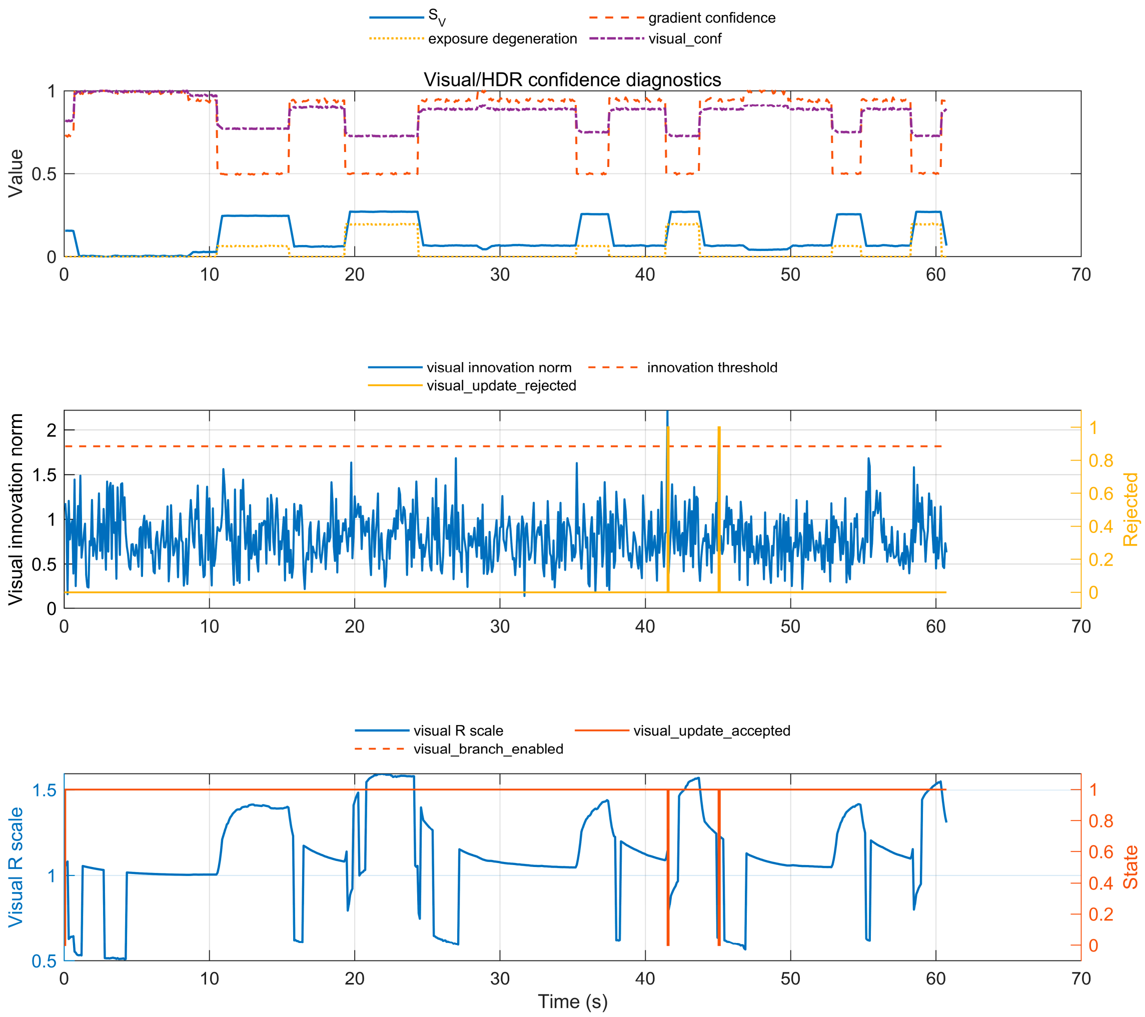

Figure S3. Visual pose-proxy confidence, normalized innovation and acceptance states. Pre-gate and applied covariance are distinct.

Supplementary Figure S4

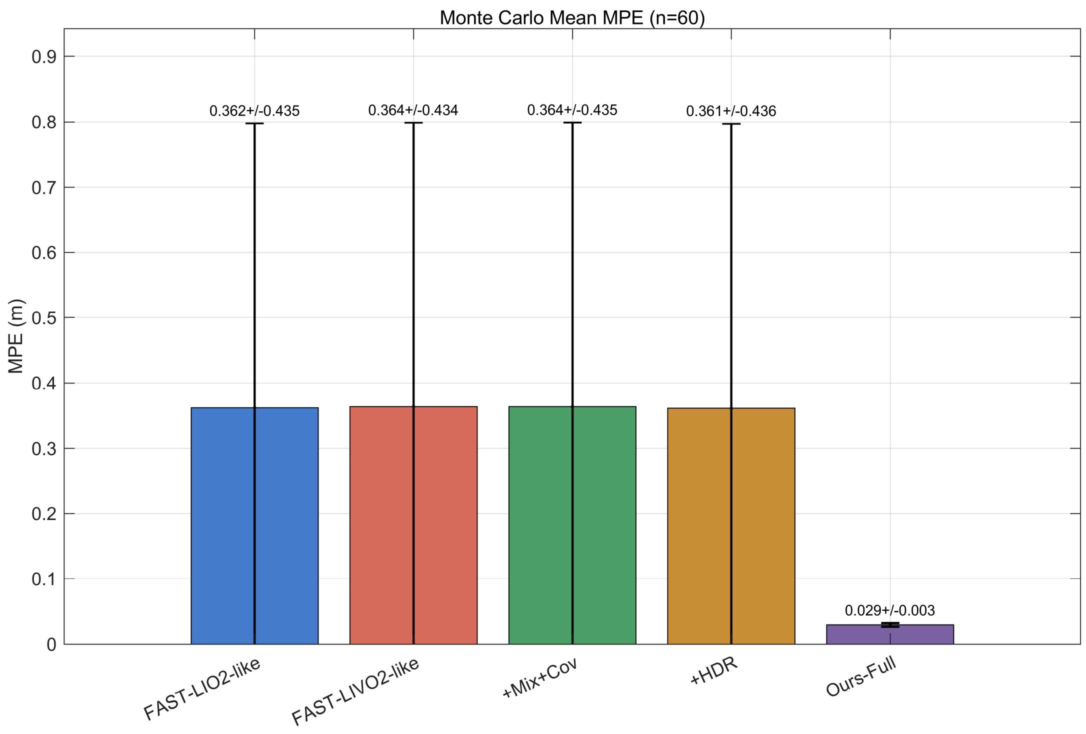

Figure S4. Mean position error (MPE) and between-trial standard deviation over all 60 trials.

Supplementary Figure S5

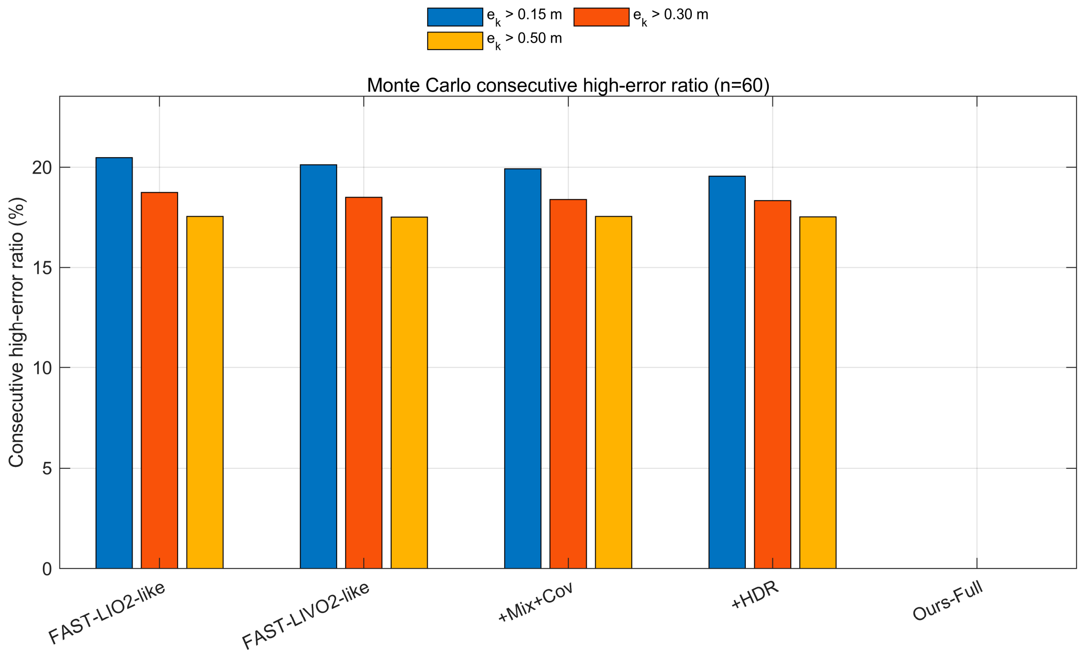

Figure S5. Consecutive high-error ratios at fixed thresholds and a five-frame event definition.

Supplementary Figure S6

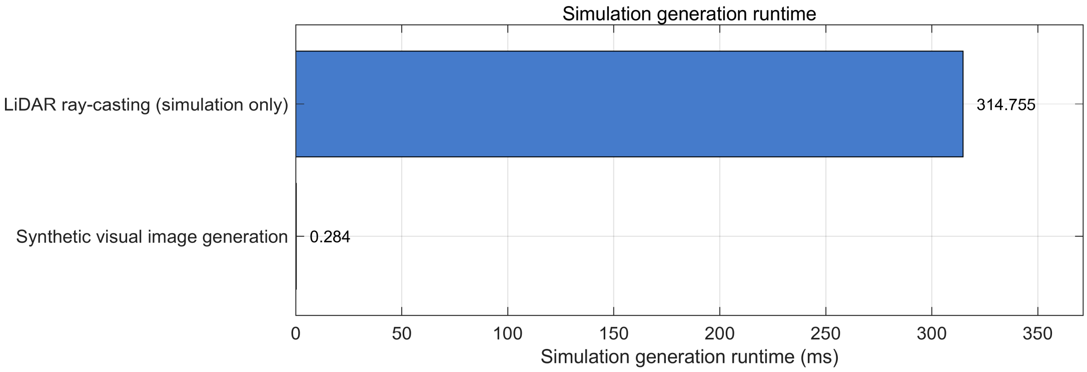

Figure S6. Synthetic scan and image generation costs, excluded from processing runtime.

Supplementary Figure S7

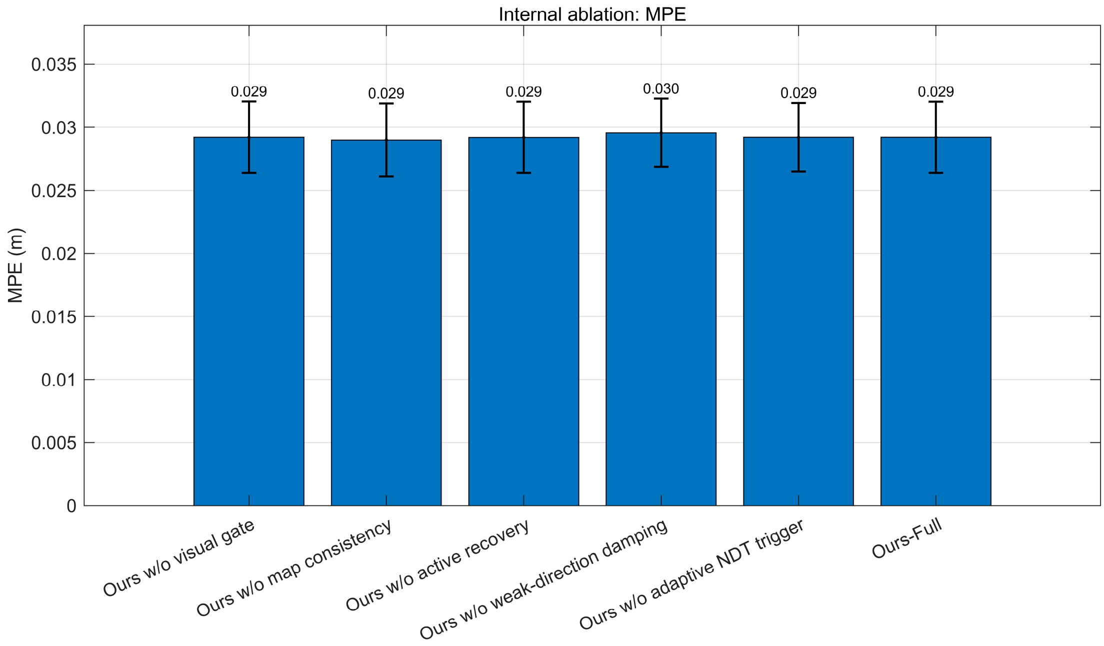

Figure S7. Internal ablation mean position error with between-trial dispersion; disabling one module need not worsen accuracy.

Supplementary Figure S8

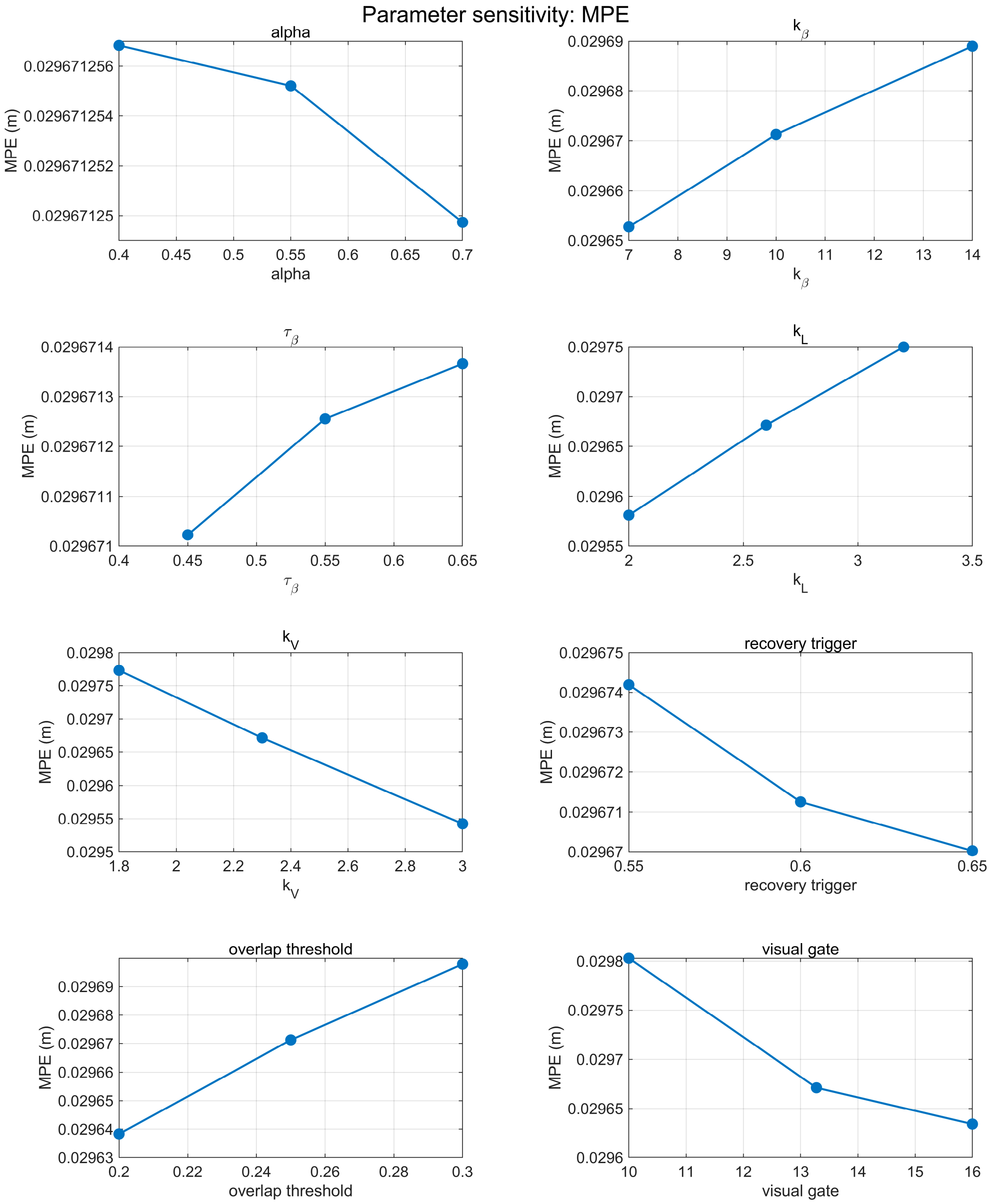

Figure S8. Single-sequence one-at-a-time sensitivity with the causal controller retained; not a Monte Carlo sensitivity claim.

Table S1. One-at-a-time sensitivity for one fixed sequence; unchanged scene and evaluation thresholds.

Parameter	Level	Mean error (m)	Peak (m)	High-error (%)	Time (ms)
alpha	0.4	0.0297	0.0957	0.00	25.74
alpha	0.55	0.0297	0.0957	0.00	25.65
alpha	0.7	0.0297	0.0957	0.00	25.59
k beta	7	0.0297	0.0957	0.00	25.54
k beta	10	0.0297	0.0957	0.00	25.48
k beta	14	0.0297	0.0957	0.00	25.47
tau beta	0.45	0.0297	0.0957	0.00	25.47
tau beta	0.55	0.0297	0.0957	0.00	25.46
tau beta	0.65	0.0297	0.0957	0.00	25.47
kL	2	0.0296	0.0957	0.00	25.47
kL	2.6	0.0297	0.0957	0.00	25.49
kL	3.2	0.0297	0.0957	0.00	25.48
kV	1.8	0.0298	0.0966	0.00	25.48
kV	2.3	0.0297	0.0957	0.00	25.46
kV	3	0.0295	0.0946	0.00	25.47
recovery trigger threshold	0.55	0.0297	0.0957	0.00	25.47
recovery trigger threshold	0.6	0.0297	0.0957	0.00	25.48
recovery trigger threshold	0.65	0.0297	0.0957	0.00	25.37
overlap threshold	0.2	0.0296	0.0957	0.00	25.49
overlap threshold	0.25	0.0297	0.0957	0.00	25.49
overlap threshold	0.3	0.0297	0.0957	0.00	25.47
visual gate threshold	10	0.0298	0.0957	0.00	25.45
visual gate threshold	13.28	0.0297	0.0957	0.00	25.55
visual gate threshold	16	0.0296	0.0957	0.00	25.47

Base Working Covariances

Table S2 specifies the diagonal base matrices in the executed four-dimensional model. Each listed scale is squared to form the corresponding diagonal entry. The fourth coordinate is converted from degrees to radians before squaring. These are tuned working covariances, not calibrated sensor uncertainty.

Table S2. Base covariance scales in position-and-yaw coordinates.

Matrix	Position scales (m)	Yaw scale (degree)
P_0	0.08, 0.08, 0.06	3.0
Q_0	0.020, 0.020, 0.015	0.8
R_p^0	0.070, 0.070, 0.050	2.0
R_n^0	0.095, 0.095, 0.070	2.8
$R_{V,\mathrm{raw}}^0$	0.080, 0.080, 0.055	2.4
$R_{V,\mathrm{HDR}}^0$	0.065, 0.065, 0.050	2.0

Runtime Distribution

Table S3 summarizes the pooled component-time sums for Ours-Full across the 60 Monte Carlo sequences. These are the original checkpoint timers, excluding data generation and archive IO. They are not sensor-to-output wall-clock latency. The archived source snapshot reproduces the measured implementation; later defensive input checks and additional covariance logging were verified to leave valid-input estimates unchanged, but their verification overhead is not substituted into these timings.

Table S3. Pooled reduced-order processing profile over 45,540 frames (frames 2-760 of each trial).

Mean (ms)	Median (ms)	P95 (ms)	Maximum (ms)
20.9089	14.9567	36.9518	281.0941

Recovery score implementation

Tables S4 and S5 document the archived implementation used for the main results. They clarify existing operations and do not introduce a new estimator or alter any reported result.

Table S4. Components of the recovery trigger score in Equation (16) of the main article. Each finite component is clipped to [0, 1] before weighted averaging. The weights below are renormalized over available components.

Component	Value before final clipping	Weight
Degeneracy	$\max(S_L, S_V, S)$	0.16
Residual	$\max(r_L, r_V)/t_r$	0.12
LiDAR innovation	$r_L/\sqrt{9.487729/4}$	0.09
Visual innovation	$r_V/\sqrt{9.487729/4}$	0.08
ICP residual	ICP RMSE divided by 0.12 m	0.08
NDT cost	NDT mean cost divided by t_N	0.08
Correspondence deficit	1 minus ICP correspondence count / 130	0.08
NDT point deficit	1 minus NDT used-point count / 40	0.08
Overlap deficit	1 minus managed overlap / 0.25	0.12
Abnormal-frame count	Consecutive abnormal frames / 3	0.07
State increment	$\sqrt{(\|\delta \mathrm{p}\|/0.20)^2+(\|\delta\psi\|/0.0698132)^2}$	0.03
Covariance trace	$\mathrm{tr}(\mathrm{P})/0.75$	0.01

r_L and r_V are the normalized residual quantities supplied by the filter to the recovery check, including any preceding robust-residual adjustment. The increment uses position in metres and yaw in radians; the implemented yaw scale is exactly 4 degrees. The covariance trace combines those coordinates without nondimensionalization and must not be interpreted as calibrated or traceable measurement uncertainty.

The residual threshold t_r is the median plus 1.2×1.4826 times the median absolute deviation (MAD), clipped to [1.4, 4.0]. It uses finite residual entries in the 40-frame causal window, before inserting the current residual; fewer than ten entries use 4.0. The recovery NDT threshold t_N uses median plus 3×1.4826 MAD over the available 30-frame cost history and is clipped to [12, 30]; an empty history uses 30.

If NDT was not executed, its cost and point-deficit weights are zero. Any nonfinite component also receives zero weight. The remaining weighted sum is divided by the sum of remaining weights. A missing NDT result is therefore not a zero-quality observation. The abnormal-frame counter is distinct from the consecutive recovery-score counter in Equation (16) of the main article.

An abnormal frame is flagged by extreme degeneracy, simultaneous hard LiDAR and visual rejection, too few NDT points or excessive NDT cost when NDT ran, a mixing weight above 0.85 with a large residual, covariance trace above 0.75, or at least three consecutive low-overlap frames. This counter resets on a normal frame. Recovery eligibility requires two consecutive scores of at least 0.60, no cooldown, and an available attempt budget; eligibility alone does not establish an accepted correction.

Extreme degeneracy means S exceeds its adaptive threshold, $S_L > 0.82$, $S_V > 0.78$, or the frontend-fallback flag is set. The adaptive threshold is the mean plus one sample standard deviation of the available 40-frame score window, clipped to [0.55, 0.88]; fewer than ten samples use 0.82. Low overlap means managed overlap below 0.25 when map-consistency checks are enabled. These operational tests do not use reference trajectory error.

Conditional NDT implementation

Table S5 specifies the executed scheduling rules. The trigger uses the pre-registration scan-geometric score, not the post-registration Hessian score or its five-frame average. The overlap proxy is ICP correspondence count divided by the original scan point count, clipped to [0, 1]; it is distinct from the managed-overlap quantity used in Table S4.

Table S5. NDT activation, caching, sampling, and iteration settings in the archived main experiment.

Operation	Executed rule
Activation	Run when the cache is invalid, scan score is at least 0.42, ICP fails, finite ICP condition number exceeds 2500, finite ICP RMSE exceeds 0.12 m, ICP correspondences fall below 130, or overlap falls below 0.08. Also run after at least two bad NDT executions if scan score exceeds 0.28 or overlap is below 0.13.
Hold counter	One trigger frame activates NDT and sets the counter to 3. Activity is checked before decrementing it. With no new trigger, NDT runs on the trigger frame and the next two frames, then stops on the third healthy frame.
Cache rebuild	Rebuild for an invalid/disabled cache, translation since the cache pose above 0.90 m, wrapped yaw change above 12 degrees, source-index change above 0.90 together with translation above 0.45 m, or finite overlap below 0.05. Otherwise reuse the cache.
Source-index change	One minus the fraction of current local-map source indices also present in the cached set; an empty set gives 1. These are subsets of the fixed known map, not demonstrated updates of an estimated SLAM map.
Voxel construction	NDT cells have side length 0.70 m; at least 4 map points are required per cell.
Input sampling	A geometry-only 0.25 m grid retains the first scan point per occupied cell. If necessary, indices are evenly selected to meet the mode budget. Material labels are not used.
Modes and budgets	Nominal: at most 220 points and 2 iterations. High degeneration (scan score >0.45): 460 points and 4 iterations. Recovery mode (bad-NDT counter at least 2): 650 points and 6 iterations; recovery mode takes precedence.
Stopping and step limits	Translation tolerance 0.002 m and yaw tolerance 0.08 degrees. Per-iteration updates are limited to 0.30 m and 8 degrees. Convergence may stop matching before the iteration budget.
Bad-NDT counter	Increment after failed NDT, fewer than 40 used points, or finite cost above the current cost gate; otherwise reset after a successful NDT execution. It is not the active-recovery score counter.
Cost gate	Use median plus 3 times 1.4826 MAD from up to 30 preceding frame costs, clipped to [12, 30]. The frontend uses 20 for an empty history, whereas the recovery-score helper uses 30.
