# Supplementary material for: A Controlled Simulation Study of Degeneracy-Aware Pose Fusion in Confined Metal Compartments
Source: Sensors (Basel). 2026 Oct 30;26(21):6873. doi: 10.3390/s26216873 (PMC13766205; doi:10.3390/s26216873)
Supplement: Supplementary file 1 [file sensors-26-06873-s001.zip › Supplementary Material S2. Controlled_Mechanism_Validation.pdf]

MATLAB Controlled Mechanism Validation

Supplementary experiments isolate the effects of robust weighting, LiDAR innovation screening, covariance adaptation and observation selection in the existing four-dimensional, known-map pose-fusion model. They are neither executions of official FAST-LIO2 or FAST-LIVO2 nor sensor-level validation in real metallic compartments.

Experimental protocol

All conditions use 60 paired trials with seeds 1001-1060. The original archived trajectories, observation noise and evaluation thresholds are retained. The default filter is re-executed on each original input and checked against the archived Full trajectory before supplementary interventions. Position errors are evaluated in the shared simulation frame without trajectory alignment. MPE denotes mean position error; RMSE denotes its root mean square.

A consecutive high-error event comprises at least five samples above 0.15, 0.30 or 0.50 m. Its ratio counts all samples in qualifying runs. Paired differences are variant minus Full, or variant minus Adaptive for the policy experiment. Percentile 95% intervals use 5000 bootstrap resamples of whole trials. They are exploratory and not adjusted for multiple comparisons; frames are not treated as independent replicates. The intervals describe noise-seed variation in the fixed simulated scene, not variation across real environments.

Attribution overview

Table SM1 reports between-trial mean and standard deviation. The added switches separate Huber weighting from the safeguards that were coupled to it in the original configuration. Invalid observations remain rejected when innovation screening is disabled. The covariance ablation disables adaptive process noise, base-covariance feedback and modality-specific scaling together; it does not isolate a single covariance parameter. Visual-confidence scaling, gate-induced inflation and map protection remain as separate safeguards.

Table SM1. Controlled ablation results over 60 paired trials.

Variant	MPE mean (SD) (m)	Peak mean (m)	High error at 0.15 m (%)
Full	0.02920 (0.00283)	0.11220	0.000
Without Huber	0.02920 (0.00283)	0.11226	0.000
Without LiDAR gate	0.34992 (0.43372)	2.10724	18.487
Without both	0.35974 (0.43612)	2.18007	19.303
Without adaptive covariance	0.02919 (0.00222)	0.09548	0.000
Fixed mixing 0.5	0.02918 (0.00282)	0.10968	0.000
Without covariance and mixing adaptation	0.02923 (0.00222)	0.09433	0.000

Conditional contributions of the safeguards

Full achieves an MPE of 0.02920 m. Removing LiDAR innovation screening changes mean MPE by 0.32072 m (paired 95% interval 0.21999 to 0.43862 m). Removing Huber weighting alone changes it by 1.58e-06 m (interval 1.12e-06 to 2.05e-06 m).

Figure SM1 separates the contribution of LiDAR screening from the other interventions. The four Huber/screening configurations also allow their conditional effects to be compared. Small or negative differences do not establish a benefit for the removed module. The large gap between the original external mechanism variants and Full should not be attributed uniformly to every adaptive component.

The difference in the Huber-removal effect between the gate-disabled and gate-enabled conditions is 0.00982 m (paired bootstrap interval 0.00806 to 0.01189 m). This interaction contrast is defined as (without both minus without gate) minus (without Huber minus Full).

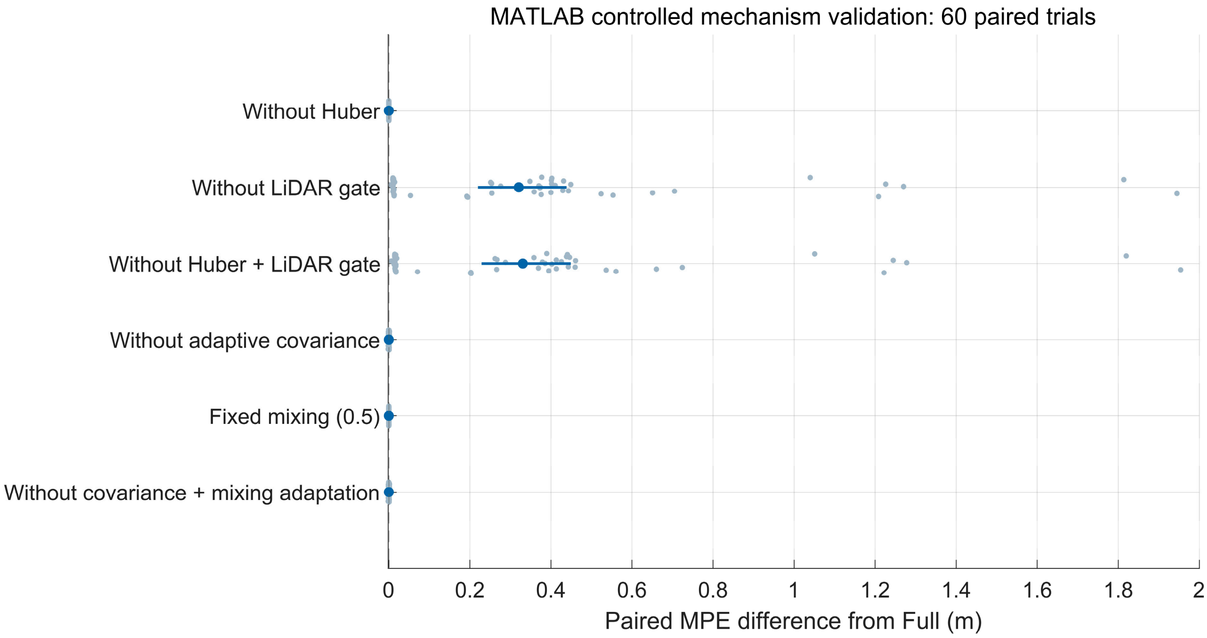

Figure SM1. Paired mean MPE differences from Full with trial-bootstrap 95% intervals. Pale dots show all 60 trial-level differences. Positive values indicate higher error after the indicated intervention. All conditions use the original observation pool.

Disabling covariance or mixing adaptation leaves the degeneracy scores available to the remaining safeguards. These variants therefore assess the indicated update rules, not the removal of all degeneracy information.

Fixed observation policies

The four policies share the same archived always-on NDT observation pool, including identical visual and inertial inputs. ICP-only and NDT-only accept only their selected source; an invalid observation is not silently replaced by the other source. Other safeguards are retained. This is a pose-observation policy comparison, not a comparison of independently executed raw-residual frontends or their computational costs.

Table SM2 and Figure SM2 summarize the paired policy results.

Table SM2. Observation-policy comparison on a common all-NDT pool.

Policy	MPE mean (SD) (m)	High error at 0.15 m (%)
Adaptive	0.02920 (0.00273)	0.000
Fixed 0.5	0.02946 (0.00271)	0.000
ICP only	0.02915 (0.00262)	0.000
NDT only	0.03460 (0.00264)	0.068

Among these policies, ICP only has the lowest mean MPE in this observation pool. The comparison does not establish universal superiority of adaptive mixing; policy selection must be interpreted together with source validity and the retained innovation safeguards.

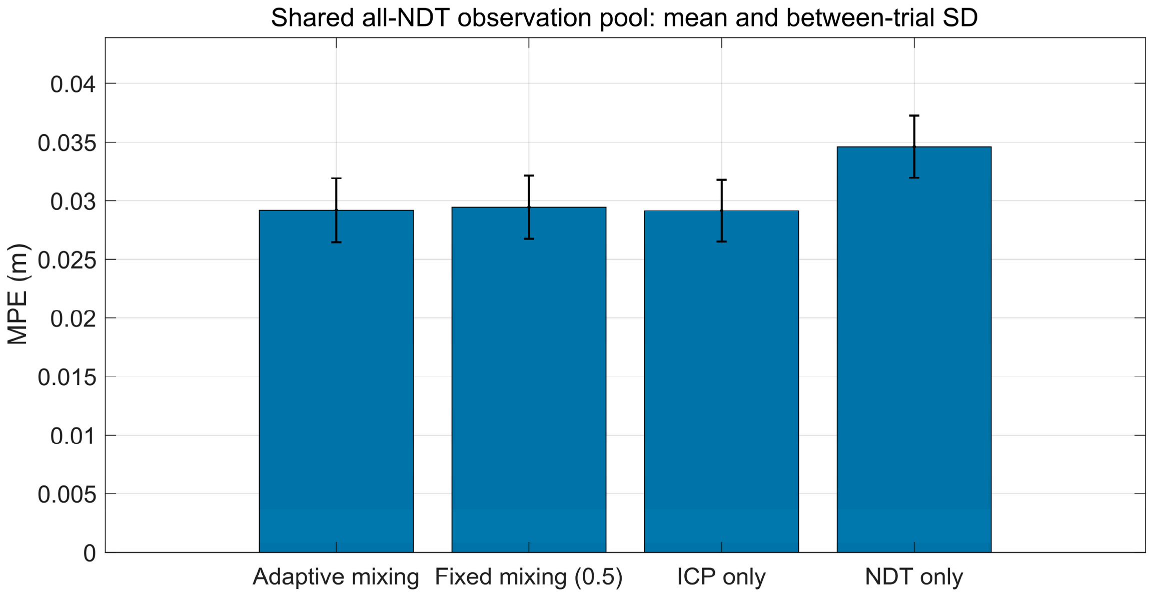

Figure SM2. Mean MPE and between-trial standard deviation for four observation policies sharing an all-NDT input pool. These results are separate from the original conditional-NDT experiment.

Additional observation corruption

The duration study adds a common pose disturbance to the LiDAR and visual observations from 20 s for 6, 12 or 24 s. The rate study uses frame-corruption probabilities of 0.05, 0.10 and 0.20, with nested masks generated from the same per-trial random sequence. The disturbance is a 0.4 m translation in a seeded random direction and a 5 degree yaw increment. Original noise is preserved; reference trajectories do not select the affected frames.

Scores, Hessians, images, scans and maps are unchanged. Thus, the intervention tests pose-observation anomalies that the existing quality indicators may miss, not newly generated geometric or photometric degeneration. Recovery still uses the original map and scans. All four variants receive exactly the same disturbed inputs. Figure SM3 shows the duration and corruption-rate responses.

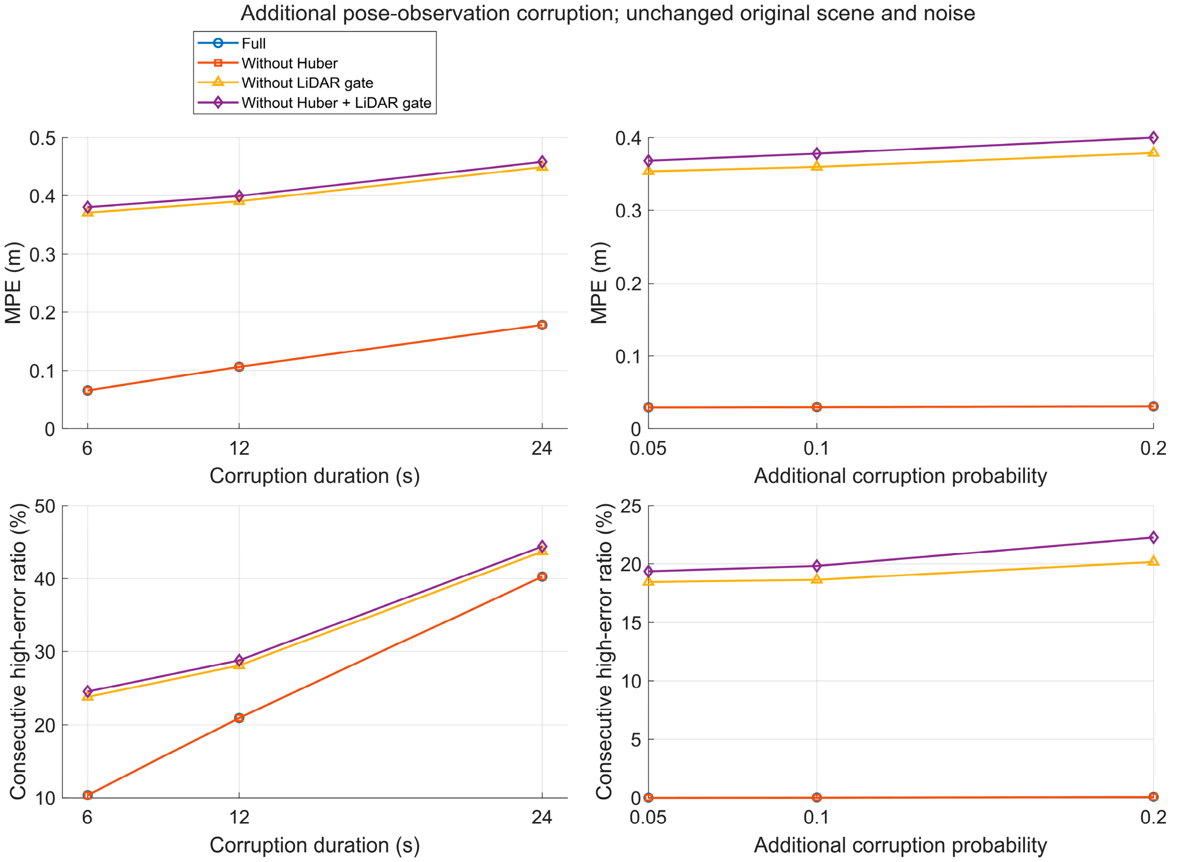

Figure SM3. Mean MPE and consecutive high-error ratio at 0.15 m under additional pose-observation corruption. Each point summarizes 60 paired trials. Masks and disturbance increments are retained.

For Full, mean MPE increases from 0.066 m at 6 s to 0.178 m at 24 s. Sustained common observation corruption therefore remains a limitation despite screening. The much smaller response to scattered corruption does not imply tolerance of prolonged invalid observations.

Processing cost and interpretation

Figure SM4 reports newly measured filter-only processing time. Variant execution order is rotated across trials. Cached frontend computation, synthetic data generation, disk loading and figure export are excluded. Runs use MATLAB R2020a on an Intel Core i7-12700KF CPU (12 cores, 20 logical processors), with serial trial execution. These timings cannot replace the original complete processing profile or establish sensor-to-output real-time latency.

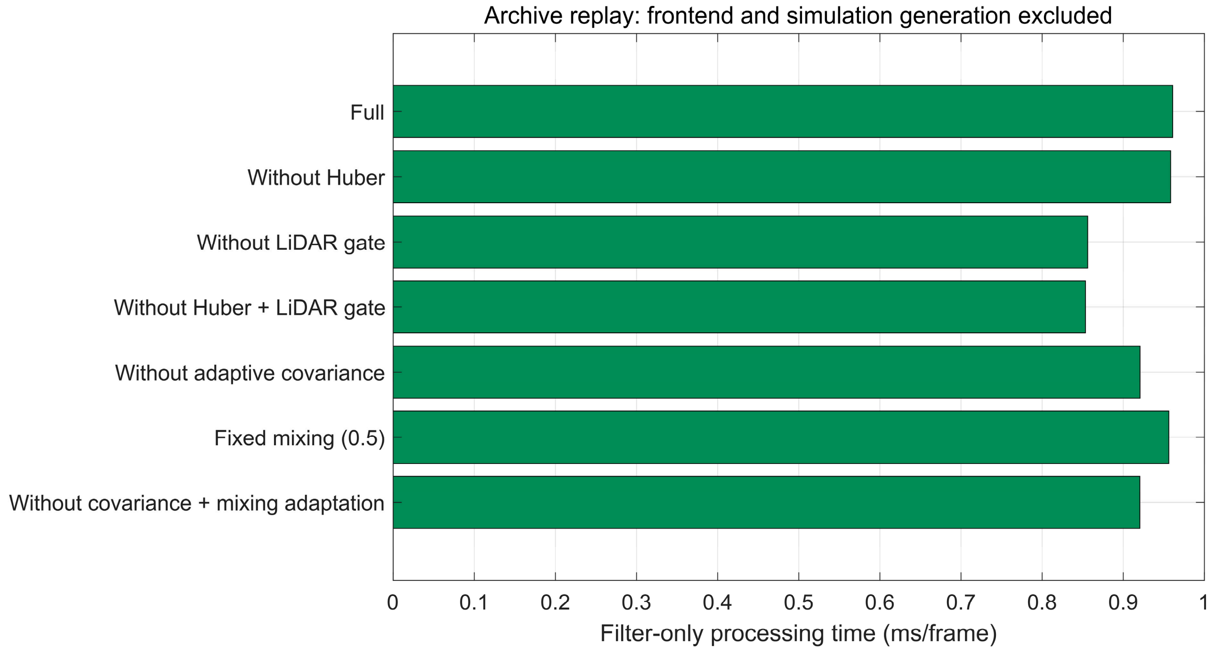

Figure SM4. Mean measured filter-only time per frame across the 60 original-input trials. Frontend processing and simulation generation are excluded.

These experiments strengthen mechanism attribution within the implemented reduced-order model. They do not validate six-degree-of-freedom raw-sensor LVI, official-system superiority, estimated-map feedback under contamination, or real-compartment trajectory accuracy. The original main results are unchanged.
