# Supplementary material for: A Controlled Simulation Study of Degeneracy-Aware Pose Fusion in Confined Metal Compartments
Source: Sensors (Basel). 2026 Oct 30;26(21):6873. doi: 10.3390/s26216873 (PMC13766205; doi:10.3390/s26216873)
Supplement: Supplementary file 1 [file sensors-26-06873-s001.zip › Supplementary Material S3. Targeted_Validation.pdf]

Supplementary Targeted MATLAB Validation

Manuscript sensors-4607454

ST1 Fixed reduced configuration

A reduced configuration was fixed before replay: adaptive covariance scaling, candidate-map protection and active recovery were disabled, and mixing was fixed at 0.5. LiDAR screening, Huber weighting, visual confidence and innovation screening, weak-direction damping, and the archived frontend inputs were retained. Across 60 paired trials, MPE was 0.029198 m for Full and 0.028923 m for Reduced. The paired difference (Reduced minus Full) was -0.000276 m, with a trial-bootstrap 95% interval of [-0.000474, -0.000081] m. This is a joint simplification, not attribution to an individual component. No non-inferiority margin was specified, so equivalence is not claimed. Reduced had lower mean error in this protocol; the additional complexity of Full is not justified by mean accuracy on these inputs. Mean trial peaks were 0.11220 m for Full and 0.09321 m for Reduced; the largest individual peaks were 0.24187 and 0.11732 m, respectively. The comparison does not test prevention of feedback-map contamination or broader operating conditions.

Table ST1. Original-input replay across 60 paired trials. Values are means unless specified. Peak is the mean per-trial maximum. Time excludes the frontend. All three high-error thresholds retain the five-consecutive-frame definition.

Variant	MPE (SD) (m)	RMSE (m)	Peak (m)	CHR 0.15 m (%)	Filter (ms)
Full	0.029198 (0.002829)	0.033859	0.112202	0.000	0.826
Reduced	0.028923 (0.002294)	0.032445	0.093207	0.000	0.424
No visual innovation gate	0.029206 (0.002837)	0.033776	0.108672	0.000	0.826

The summary includes maximum trial values and paired 95% bootstrap intervals using 5000 resamples of 60 whole trials. Intervals are unadjusted across comparisons. The filter replay order rotates across trials; the shared frontend is not rerun.

ST2 Visual-only pose corruption

The visual-specific comparison retains the original LiDAR observations, propagation inputs, quality indicators and scene. Only synthetic visual pose observations receive the pre-specified additional translation of 0.4 m in one fixed random direction per trial and yaw of 5 degrees. Corruption lasts 6, 12 or 24 s from 20 s, or uses nested random masks with probabilities of 0.05, 0.10 and 0.20. Each condition has 60 paired trials. The control disables only visual innovation rejection and its soft covariance inflation; confidence-based covariance scaling, low-confidence rejection and invalid-input rejection remain enabled. Innovation-dependent adaptive covariance evolution is also retained. Thus this is not a comparison with an entirely unprotected visual branch.

Table ST2. Mean error and consecutive high-error ratio at 0.15 m. Delta is No visual innovation gate minus Full; the interval is a paired 95% bootstrap interval for MPE difference.

Condition	Full MPE (m)	No gate MPE (m)	Delta [95% CI] (m)	CHR Full / No gate (%)
6 s	0.05029	0.06308	0.01278 [0.00881, 0.01674]	5.897 / 9.730
12 s	0.07383	0.09984	0.02601 [0.01732, 0.03464]	12.197 / 19.647
24 s	0.11681	0.17267	0.05586 [0.03879, 0.07300]	23.963 / 39.406
5%	0.02912	0.03155	0.00243 [0.00215, 0.00272]	0.000 / 0.000
10%	0.02919	0.03472	0.00554 [0.00489, 0.00621]	0.000 / 0.127
20%	0.02946	0.04486	0.01540 [0.01372, 0.01721]	0.020 / 0.680

At corruption probabilities of 0.05, 0.10 and 0.20, Full produced MPE values of 0.02912, 0.02919, 0.02946 m, compared with 0.03155, 0.03472, 0.04486 m without the visual innovation gate. Under persistent corruption lasting 6, 12 and 24 s, the corresponding Full values were 0.05029, 0.07383, 0.11681 m, versus 0.06308, 0.09984, 0.17267 m without the gate. Tables ST2-ST3 report rejection proportions and paired intervals. Rejection of an injected observation tests compatibility with the evolving prediction, not detection of its true cause. Sustained bias can remain compatible as the estimate and working covariance evolve. These results concern a pose proxy, not pixel-level HDR alignment.

Although screening reduces aggregate error in these added conditions, Full still reaches a mean consecutive high-error ratio of 23.96% at 24 s. Removing the gate gives lower MPE in 15 of the 60 trials at that duration. Screening therefore does not guarantee improvement for every realization or eliminate sustained biased updates.

Table ST3. Full rejection percentages averaged over trials, with each trial using its injected or uninjected frame count as denominator. Frame 1 is excluded. Uninjected does not mean error-free. Reason flags may overlap and should not be summed.

Condition	Injected rejected (%)	Uninjected rejected (%)
6 s	49.11	1.32
12 s	43.79	1.72
24 s	41.10	1.64
5%	99.38	0.20
10%	99.31	0.21
20%	99.14	0.18

Figure ST1 is presented below.

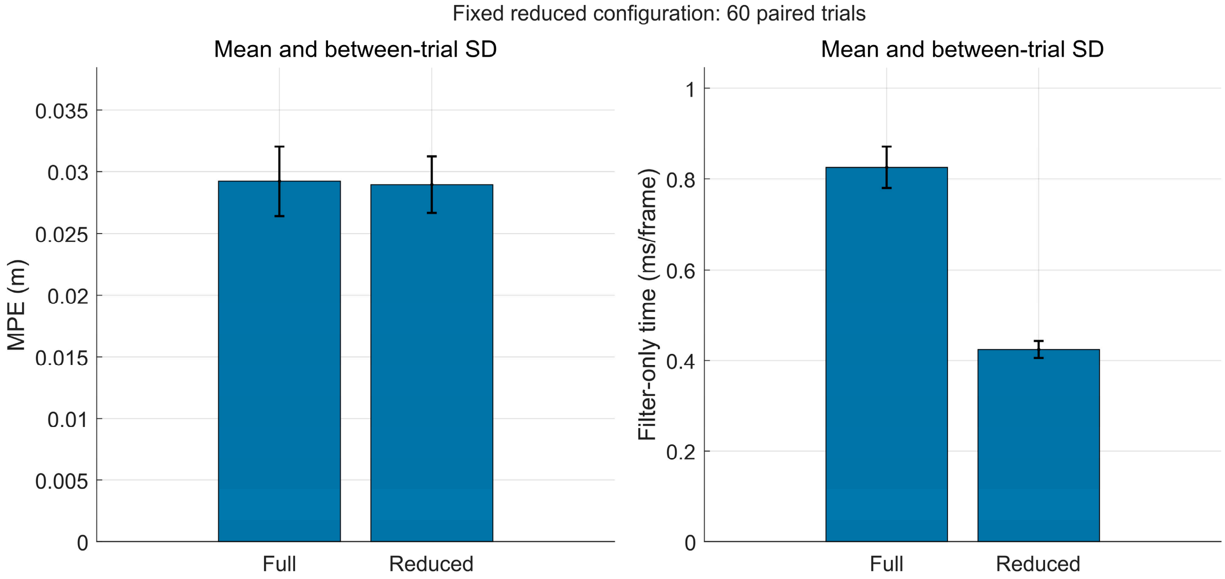

Figure ST1. Fixed reduced configuration versus Full. Bars show means and between-trial standard deviations; replay cost excludes all frontend computation. The joint reduction cannot isolate the contribution of a single removed component.

Figure ST2 is presented below.

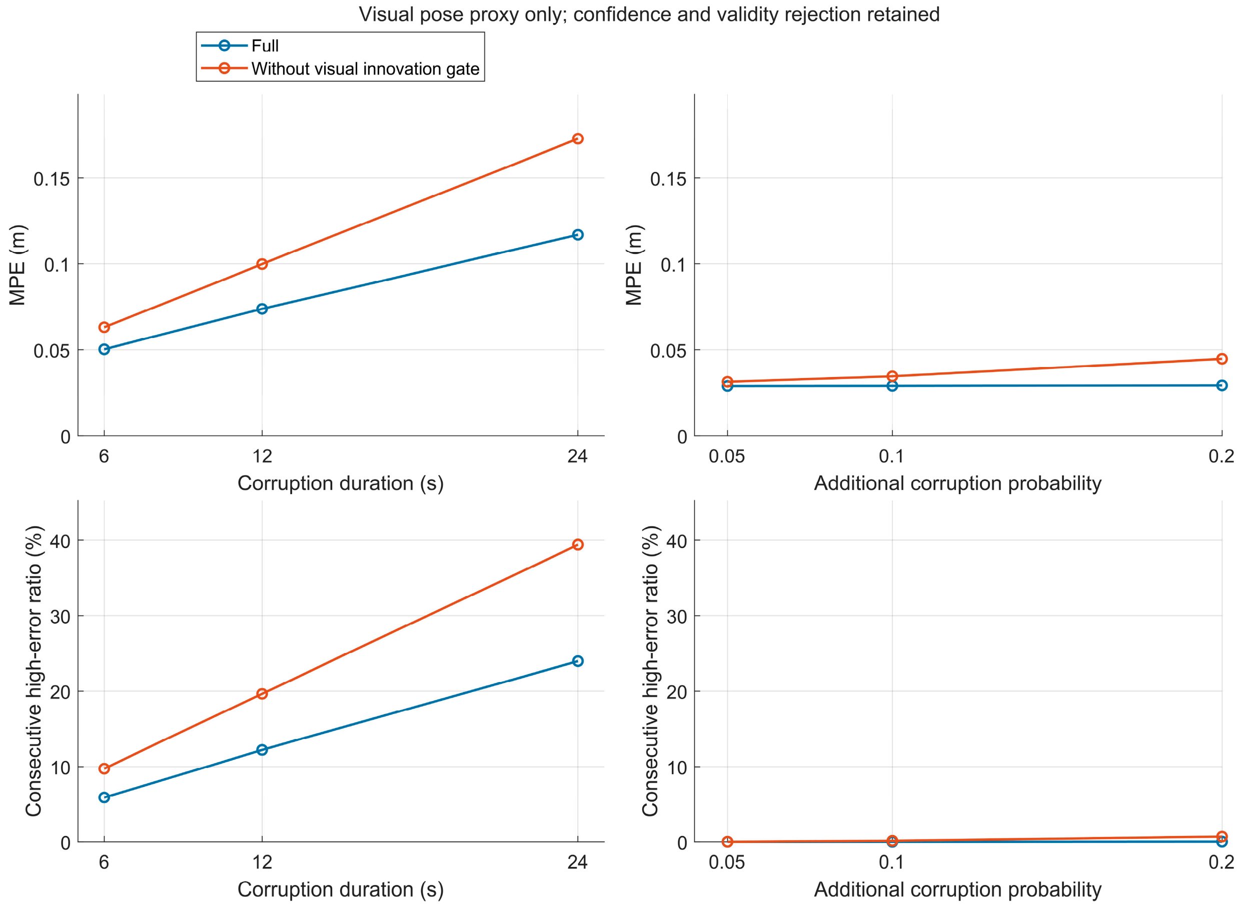

Figure ST2. Visual-only synthetic pose corruption with and without innovation screening. Confidence and invalid-input rejection remain enabled. Mean error and consecutive high-error ratio are shown separately for sustained and randomly scattered corruption; lines connect the prescribed test levels.

Figure ST3 is presented below.

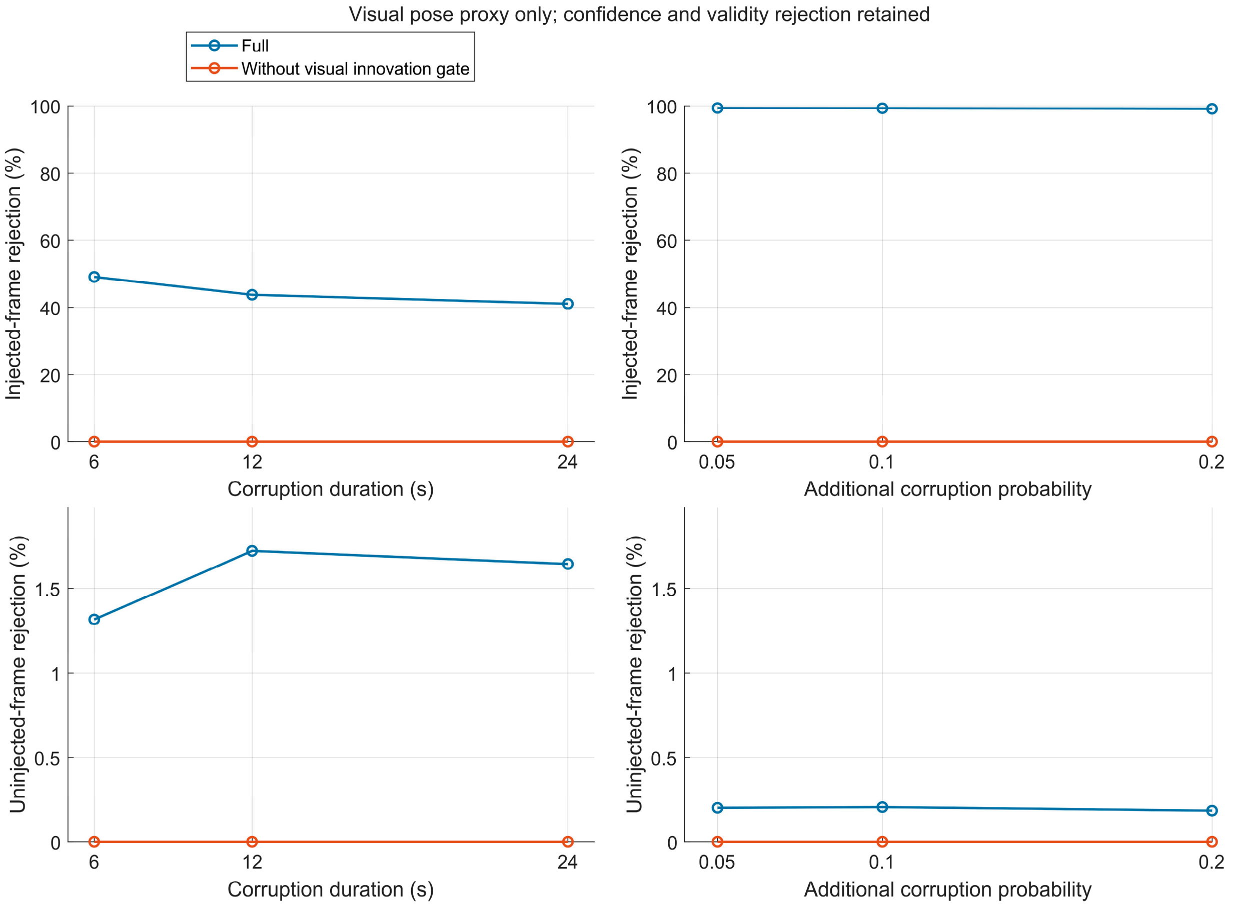

Figure ST3. Mean trial-wise rejection percentages. Injected and uninjected frames have separate denominators. The latter are not clean negative examples, and the plot should not be interpreted as a false-positive-rate analysis.
