# Supplementary material for: A Controlled Simulation Study of Degeneracy-Aware Pose Fusion in Confined Metal Compartments
Source: Sensors (Basel). 2026 Oct 30;26(21):6873. doi: 10.3390/s26216873 (PMC13766205; doi:10.3390/s26216873)
Supplement: Supplementary file 1 [file sensors-26-06873-s001.zip › Supplementary Material S4. Transfer_and_Recovery_Validation.pdf]

Supplementary Transfer and Recovery Validation

Manuscript sensors-4607454

SV1 Frozen parameter transfer

Two additional conditions were fixed before execution, without retuning the estimator. The first reverses traversal of the original 18 by 8 by 5.2 m cabin and shifts yaw by pi. The second uses a 22 by 6.4 by 5.2 m cabin with the original normalized route generator, changing both geometry and physical trajectory. Each condition has 60 paired trials, using new seeds 2001-2060 and 3001-3060, respectively. All 760 samples, noise settings, quality rules, evaluation thresholds and estimator parameters are retained. Scans, image-quality inputs and registration are regenerated; Full and the previously fixed Reduced configuration then receive identical inputs.

Reverse traversal gives MPE of 0.029198 m for Full and 0.029413 m for Reduced. The paired difference (Reduced minus Full) is 0.000214 m, with a 95% whole-trial bootstrap interval [0.000052, 0.000379] m. Full has lower mean error in this condition.

The resized cabin gives MPE of 0.029935 m for Full and 0.029207 m for Reduced. The paired difference (Reduced minus Full) is -0.000728 m, with a 95% whole-trial bootstrap interval [-0.000892, -0.000573] m. Reduced has lower mean error in this condition.

Table SV1. Means across 60 paired trials per condition. MPE and RMSE are distinct metrics. Peak denotes the mean per-trial maximum; CHR uses 0.15 m and at least five consecutive samples.

Condition	Variant	MPE (m)	RMSE (m)	Peak (m)	CHR (%)
Reverse	Full	0.02920	0.03376	0.10804	0.00000
Reverse	Reduced	0.02941	0.03313	0.09899	0.00000
Resized	Full	0.02994	0.03470	0.12529	0.00000
Resized	Reduced	0.02921	0.03276	0.09806	0.00000

Both mean-error differences are below 1 mm, with opposite signs across conditions. Intervals excluding zero therefore do not establish a practically important or uniform advantage for Full. Table SV1 and Figure SV1 report the two conditions separately. These are prescribed transfers within the same synthetic model, not broad environmental generalization. The resized case does not isolate geometry from trajectory, and reversing a path does not create an independent environment. Intervals use 5000 whole-trial resamples and are not adjusted for multiple comparisons. No configuration is selected or tuned from these outcomes.

SV2 Recovery under matched disturbances

Recovery-enabled and recovery-disabled configurations are compared on all 60 original archived trials. Only the active-recovery switch differs; rejected recovery attempts can also affect subsequent covariance in the enabled configuration. The original-input replay is supplemented by 6, 12 and 24 s disturbances starting at 20 s. A fixed random translation of 0.4 m and a yaw offset of 5 degrees are added either to LiDAR pose observations alone or jointly to LiDAR and visual pose observations. Paired configurations use identical disturbances. Scans, the known map, quality diagnostics and prediction inputs are unchanged. The existing four-attempt budget, cooldown and acceptance thresholds remain fixed; reference errors are used only for evaluation.

Table SV2. Enabled/disabled recovery means. Delta is disabled minus enabled MPE with a paired 95% whole-trial bootstrap interval. The original-input control is included; all conditions retain 60 trials.

Condition	MPE on/off (m)	Delta [95% CI] (m)	CHR on/off (%)
Original	0.02920 / 0.02919	-0.000008<br>[-0.000011, -0.000006]	0.000 / 0.000
LiDAR 6 s	0.02964 / 0.02963	-0.000008<br>[-0.000011, -0.000005]	0.000 / 0.000
LiDAR 12 s	0.03023 / 0.03022	-0.000010<br>[-0.000015, -0.000006]	0.000 / 0.000
LiDAR 24 s	0.03391 / 0.03390	-0.000010<br>[-0.000015, -0.000006]	0.421 / 0.421
Common 6 s	0.06617 / 0.06626	0.000089<br>[0.000016, 0.000168]	10.342 / 10.360
Common 12 s	0.10650 / 0.10648	-0.000027<br>[-0.000064, 0.000000]	20.934 / 20.928
Common 24 s	0.17811 / 0.17813	0.000026<br>[-0.000030, 0.000085]	40.252 / 40.261

For disabled-minus-enabled MPE, 1 of the six corruption-condition intervals lie above zero, 3 lie below zero and 2 include zero. The condition-specific results, rather than the existence of the recovery routine, determine the supported benefit. All absolute mean-error differences are below 0.1 mm. Recovery remains an optional safeguard rather than a primary contribution. An accepted geometric improvement is not guaranteed to reduce position error.

SV3 Recovery decisions and settling

Across the seven conditions, the enabled configuration makes 1397 attempts and accepts 9 corrections in 420 trial-condition executions. Of these accepted corrections, 6 increase immediate reference position error; the maximum accepted translation is 0.17135 m. These totals include repeated seeds under different conditions and are not 420 independent trajectories. In the six corruption conditions, 540 attempts occur before the added disturbance, and 12 of 360 trial-condition executions have already exhausted the four-attempt budget by 20 s.

Table SV3. Recovery-enabled totals across 60 trials per condition. Error-increasing accepts compare immediate position error before and after the bounded correction, using reference positions only offline.

Condition	Attempts	Accepted	Rejected	Error increased
Original	196	1	195	1
LiDAR 6 s	182	1	181	1
LiDAR 12 s	200	2	198	2
LiDAR 24 s	193	2	191	2
Common 6 s	227	1	226	0
Common 12 s	196	1	195	0
Common 24 s	203	1	202	0

Table SV4. Post-disturbance settling. Values are enabled/disabled. Unsettled trials are counted explicitly. Restricted time equals settling time if observed, otherwise elapsed follow-up from the first post-disturbance timestamp to the final timestamp. Its mean is not an uncensored recovery-time estimate. High-error duration uses qualifying sample counts times 0.08 s; settling uses timestamp differences. Follow-up differs by disturbance duration.

Condition	Longest run (s)	Unsettled /60	Restricted time (s)	Follow-up (s)
LiDAR 6 s	0.00 / 0.00	0 / 0	0.00 / 0.00	34.72
LiDAR 12 s	0.00 / 0.00	0 / 0	0.00 / 0.00	28.72
LiDAR 24 s	0.26 / 0.26	0 / 0	0.05 / 0.05	16.72
Common 6 s	6.29 / 6.30	0 / 0	0.88 / 0.89	34.72
Common 12 s	12.73 / 12.72	0 / 0	1.32 / 1.32	28.72
Common 24 s	24.47 / 24.48	0 / 0	1.06 / 1.07	16.72

Tables SV2–SV4 and Figures SV2–SV3 report error, qualifying high-error duration, acceptance decisions and post-disturbance settling. Settling is the first five-sample run at or below 0.15 m after disturbance removal; trials that do not settle are censored, not assigned zero time. Healthy scans and a fixed known map are retained during pose corruption, so this does not validate recovery from corrupted scans, map contamination or sensor loss. Error-increasing acceptance is assessed offline and is not part of the acceptance test. Parallel-run timings are not used to revise the main runtime measurements.

Figure SV1 is presented below.

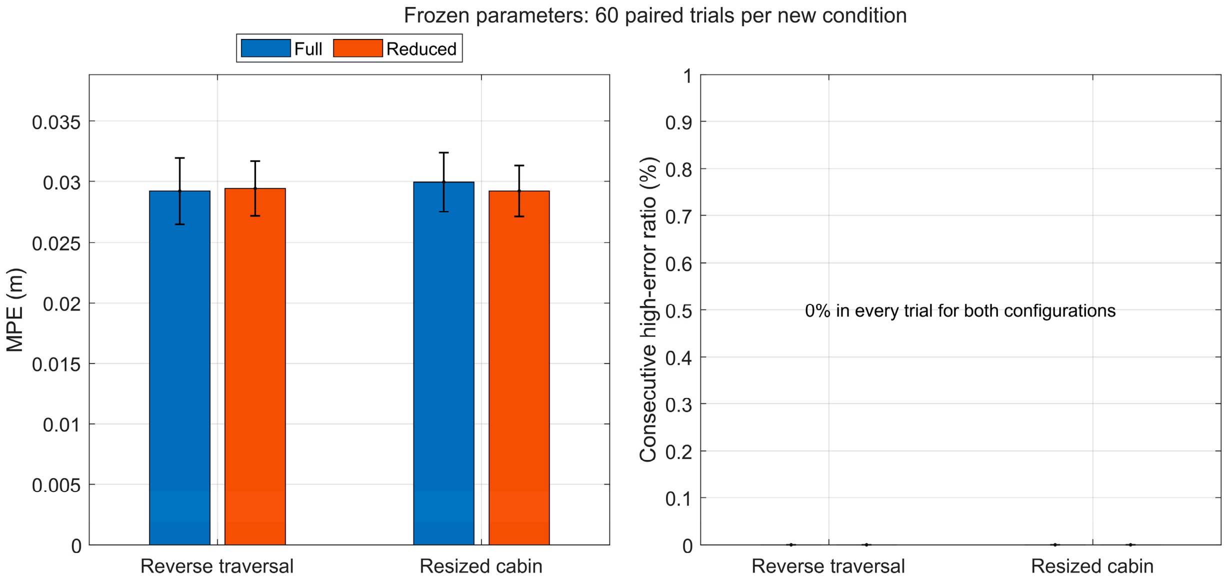

Figure SV1. Full and the fixed Reduced configuration under two new synthetic conditions. Bars and error bars show means and between-trial standard deviations, not confidence intervals. Both conditions retain the original estimator parameters.

Figure SV2 is presented below.

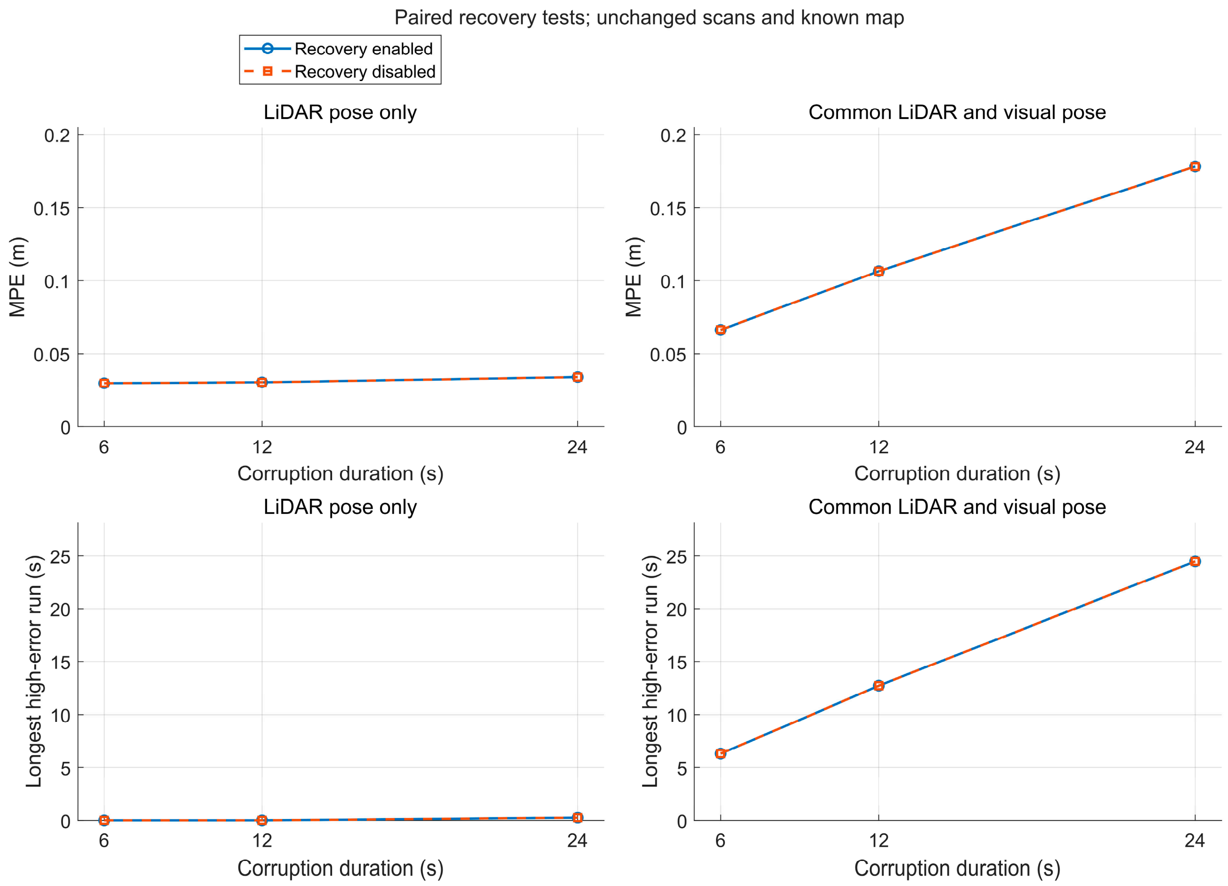

Figure SV2. Paired recovery enabled/disabled comparisons under LiDAR-only and common pose corruption. Points show means across 60 trials; lines connect the prescribed durations. Raw scans and the known map remain unchanged.

Figure SV3 is presented below.

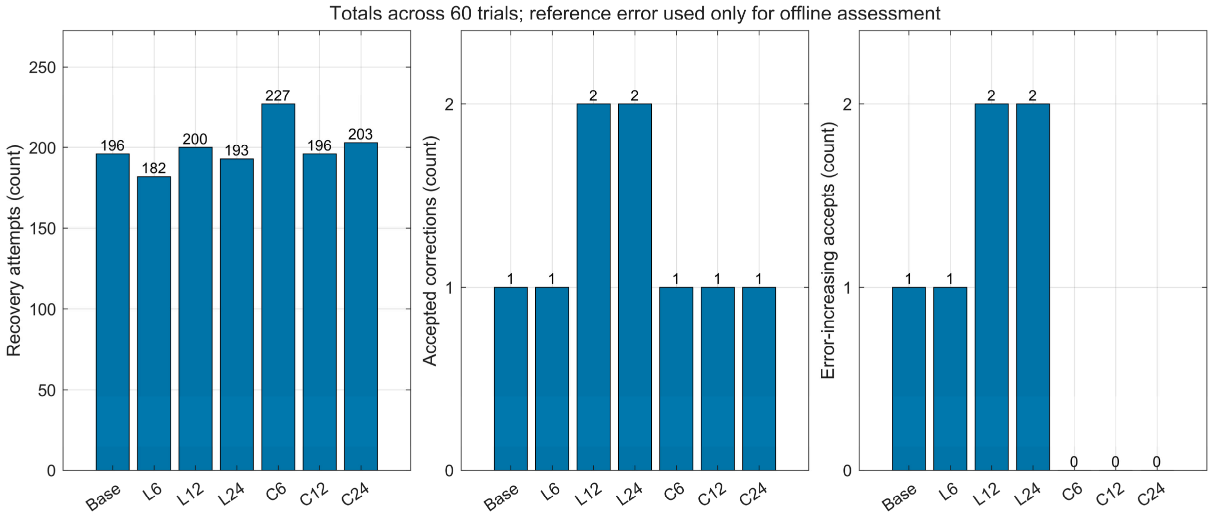

Figure SV3. Recovery decisions summed across 60 trials per condition. Base denotes original inputs; L and C denote LiDAR-only and common corruption, followed by duration in seconds. Error-increasing acceptance is an offline diagnostic, not a trigger or an independent failure label.
